# Identification of astroglia-like cardiac nexus glia that are critical regulators of cardiac development and function

**DOI:** 10.1371/journal.pbio.3001444

**Published:** 2021-11-18

**Authors:** Nina L. Kikel-Coury, Jacob P. Brandt, Isabel A. Correia, Michael R. O’Dea, Dana F. DeSantis, Felicity Sterling, Kevin Vaughan, Gulberk Ozcebe, Pinar Zorlutuna, Cody J. Smith

**Affiliations:** 1 Department of Biological Sciences, University of Notre Dame, Notre Dame, Indiana, United States of America; 2 Center for Stem Cells and Regenerative Medicine, University of Notre Dame, Notre Dame, Indiana, United States of America; 3 Department of Aerospace and Mechanical Engineering, University of Notre Dame, Notre Dame, Indiana, United States of America; University of Pittsburgh, UNITED STATES

## Abstract

Glial cells are essential for functionality of the nervous system. Growing evidence underscores the importance of astrocytes; however, analogous astroglia in peripheral organs are poorly understood. Using confocal time-lapse imaging, fate mapping, and mutant genesis in a zebrafish model, we identify a neural crest–derived glial cell, termed nexus glia, which utilizes Meteorin signaling via Jak/Stat3 to drive differentiation and regulate heart rate and rhythm. Nexus glia are labeled with *gfap*, *glast*, and *glutamine synthetase*, markers that typically denote astroglia cells. Further, analysis of single-cell sequencing datasets of human and murine hearts across ages reveals astrocyte-like cells, which we confirm through a multispecies approach. We show that cardiac nexus glia at the outflow tract are critical regulators of both the sympathetic and parasympathetic system. These data establish the crucial role of glia on cardiac homeostasis and provide a description of nexus glia in the PNS.

## Abstract 2

Do astrocyte-like cells exist in the peripheral nervous system? This study reveals that astrocyte-like cells termed cardiac nexus glia populate the heart, and that these cells are important for cardiac homeostasis, modulating heart rate and rhythm during development.

## Introduction

Throughout the vertebrate nervous system, glial cells are developmentally and functionally essential [1–3]. The existence of diverse populations of glia allows for integral support to neurons, including modulation of neuronal development [2], signal propagation [4], synaptic support [5], metabolic aid [6], and phagocytic clearance of debris [7]. These roles are divided by the specialized glial populations, largely segregated into ensheathing glia, astroglia, and microglia [4,8,9]. These glia are further divided into cell types based on their central (CNS) or peripheral (PNS) nervous system residence. As the roles for each of the cell types expands in the CNS, our understanding of specialized glia remains a major barrier to understanding the full functionality of the PNS, especially in the context of organ function.

The PNS is an integrated network of nerves that control everyday health [10,11]. In addition to the widely studied nerve system that controls skeletal muscle movement, the PNS has profound control over every organ [12]. This control is enacted through a hierarchy of neuronal processes, with the PNS extending parasympathetic and sympathetic neurons to regulate the autonomic nervous system (ANS) responses known as the “rest-and-digest” and “fight-or-flight” responses, respectively [13]. These neurons then synapse with internal nerve networks that are specialized to each organ, which allows for fast modulation of resident organ-specific cell types to maintain homeostasis [14,15]. For example, this hierarchy is especially critical to the heart, which must constantly maintain a rhythmic beating for blood circulation [16]. The internal nerve network of the heart is known as the intracardiac nervous system (ICNS) and modulates either cardiomyocytes or the ANS to tightly control heart rate and rhythm [14,17,18]. While the role of neurons in organ control has been characterized, current research is lacking an understanding of glial involvement.

In analogous complex networks that are present in the brain, astroglia populations aid in the integration process between neurons [19–24]. Similarly, enteric glia express common astroglia markers and function in the gut by controlling neuronal synapses or through direct interactions with intestinal muscle [25–27]. Satellite glia have also been described as glial cells of the PNS that regulate neuronal activity. These cells have primarily been researched in the context of somatosensory neurons [28]. However, other astroglia-like cell types localized within organs have yet to be identified and characterized. This leaves current models of PNS construction and function lacking a critical cell type in the nervous system. While it seems possible astroglia do not extensively populate organs, there have been reports of glial-like cells in the spleen [29], pancreas [30], lungs [31], and skin [32], and electron microscopy studies have identified glial-like processes in the heart [33]. Together, these data support the conclusion we may have yet to uncover important glial-like cell types.

Recent work has described cells in the adult heart that respond to injury after catheter ablation treatment for atrial fibrillation and express the astroglial marker, *s100b*, underscoring the importance of potential glial populations in the heart [34]. Additionally, the developing heart is seeded by an underdescribed population of neural crest cells at the outflow tract (OT) [35,36], which is intriguing, given that most peripheral glial populations are neural crest derived [10]. These cells are required for formation, remodeling, and blood vessel patterning of the OT and are highly associated with congenital heart disease (CHD), given that OT abnormalities account for 30% of CHD morbidity and mortality [37–40]. Despite the essential role these cells have in cardiac development, the identity of this population remains a mystery. Taken together, more research is needed to understand the presence, development, and primary function of glia within the cardiac system.

Using analysis from zebrafish, mouse, and human tissue, we searched for astroglial-like cells in the heart. Here, we describe a population of *gfap*^*+*^ glia in the heart with integral roles in cardiac development and function. Analysis of zebrafish, mouse, and human tissue reveals conservation of this *gfap*^*+*^ population between species. Further analysis of published single-cell sequencing of human embryonic and mouse adult hearts also reveals an abundant up-regulation of glial-associated genes. Focusing on the *gfap*^*+*^ population in zebrafish, we discovered that these cells arise from the hindbrain neural crest and migrate to the heart, where they utilize Meteorin signaling through Jak/STAT3 to drive differentiation. Once present, cardiac glia inhibit neuronal innervation and regulate heart rate and rhythm via sympathetic and parasympathetic regulation and additionally show that OT-located cardiac glia control heart rate. In the adult, these cells localize with neurons in a net-like morphology and interact with synapses. Due to their morphology and glial-like molecular identity, we have termed these *gfap*^*+*^ cells cardiac nexus glia (CNG). Together, these data provide evidence of a new peripheral glial population that regulates cardiac function.

## Results

### Cardiac *gfap*^*+*^ cells are conserved across species and have astroglial properties

**Fig 1:**
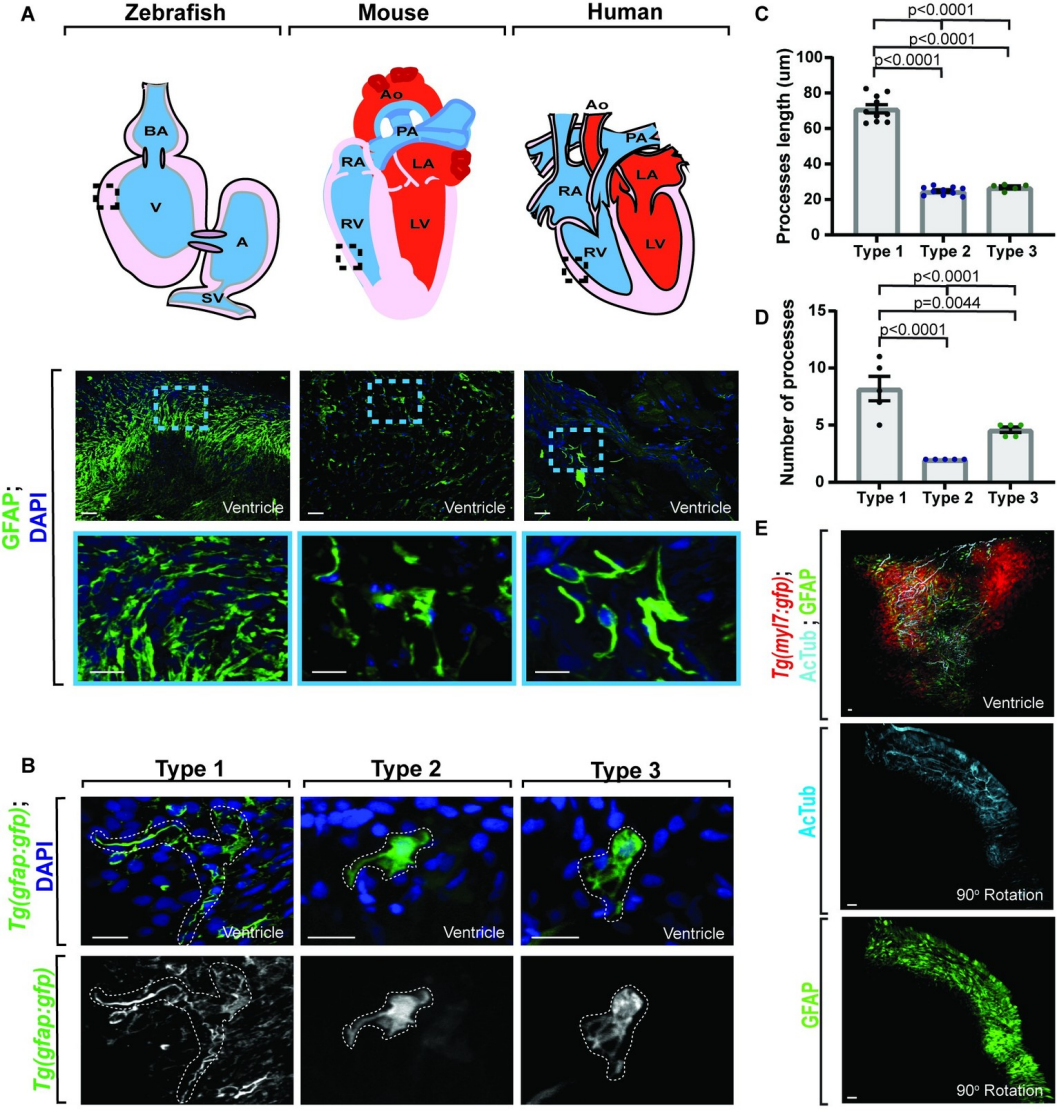
Adult *gfap*^*+*^ cells are conserved across species. (A) Confocal maximum z-projection from adult zebrafish, mouse, and human ventricle stained with GFAP. Schematics of hearts per species (above) indicate location of imaging. G*fap*^*+*^ cells are present across species. (B) Confocal 10 μm z-projection of adult *Tg(gfap*:*gfp)* ventricles with 3 distinct morphologies of *gfap*^*+*^ cells. (C) Quantification of process length between *gfap*^*+*^ cell types. (D) Quantification of process number per *gfap*^*+*^ cell type. (E) Confocal maximum z-projection from adult *Tg(myl7*:*gfp)* ventricles stained with AcTub and GFAP showing localization. G*fap*^*+*^ cells localize with *AcTub*^*+*^ axons. Data are represented as mean ± SEM. Scale bar equals 10 μm. Statistics summarized in S1 Table. See S1 Data for raw data. A, atrium; AcTub, acetylated tubulin; AO, aorta; BA, bulbous arteriosus; LA, left atrium; LV, left ventricle; PA, pulmonary artery; RA, right atrium; RV, right ventricle; SV, sinoatrial valve; V, ventricle.

Specialized glial populations are integral to nervous system function [1–3]. Seeking additional specialized glial cells in the periphery, we first screened adult zebrafish ventricles for cells that express the astroglial gene, *gfap*. Screening of adult zebrafish hearts demonstrated an abundant population of *gfap*^+^ cells (**Fig 1A**). Further analysis of mouse and human ventricle tissue also revealed morphologically similar *GFAP/Gfap*^*+*^ cells (**Fig 1A**), consistent with the idea that this population could be conserved across species. Using the zebrafish heart as a model, we additionally characterized 3 distinct morphologies associated with the *gfap*^*+*^ cells, which we refer to as Type 1 to 3 (**Fig 1B**). The majority (72.90%) of *gfap*^*+*^ cells belonged to the Type 1 category and had an average of 8.20 ± 1.07 processes with an average length of 71.15 ± 2.34 μm (**Fig 1C and 1D)** that encompassed the heart (*n =* 8 hearts) in a net-like pattern. Conversely, Type 2 cells had an average of 2.00 ± 0.00 processes (*n =* 8 hearts) with an average length of 24.75 ± 0.72 μm (*n* = 5 hearts) (**Fig 1C and 1D),** while Type 3 cells had an average of 4.60 ± 0.24 processes (*n* = 8 hearts) that were an average length of 26.86 ± 0.79 μm (*n* = 5 hearts) (**Fig 1C and 1D**). Given that Type 1 cells have long, extensive processes, we next asked if those processes were located in an area to potentially impact cardiac ganglia. We therefore asked if the *gfap*^*+*^ cells localize to neuronal axons in the heart. To address this, we used a *Tg (myl7*: *gfp)* adult heart, which uses regulatory regions of *myl7* to label cardiomyocytes. We then stained the heart with the axonal marker, acetylated tubulin (AcTub) [41], and GFAP. This revealed a close association between GFAP^*+*^ processes and AcTub^*+*^ axons (**Fig 1E**), suggesting that these cells are located in an area to potentially impact neurons in the heart.

**Fig 2:**
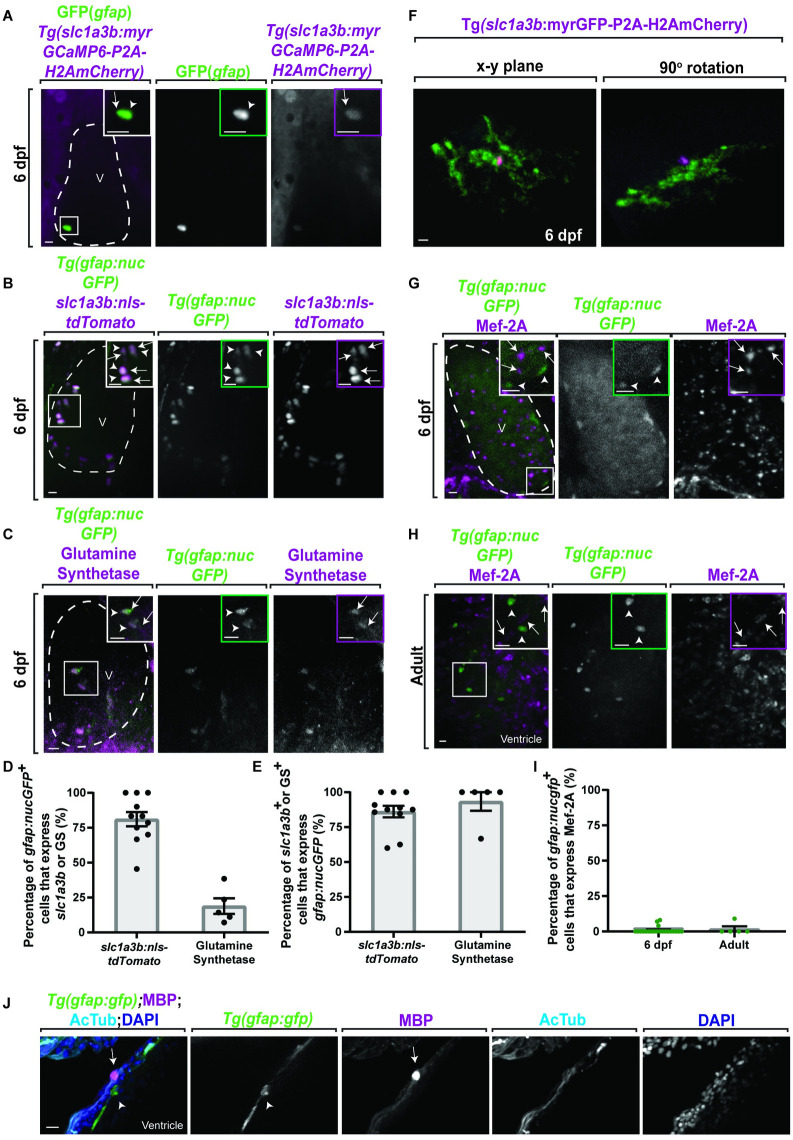
*gfap*^*+*^ cells express the astroglial gene *slc1a3b* but not cardiomyocyte genes. (A) Confocal 20 μm z-projection of 6 dpf *Tg(gfap*:*nucGFP);Tg(slc1a3b*:*myrGcAMP6-P2A-H2AmCherry)* ventricle stained with GFP. GFP^*+*^ cells (white arrowhead) colocalize with H2AmCherry^*+*^ cells (white arrow). (B) Confocal 20 μm z-projection of 6 dpf *Tg(gfap*:*nucGFP)* ventricle injected with *slc1a3b*:*nls-tdTomato*. nucGFP^***+***^ cells (white arrowheads) colocalize with tdTomato^***+***^ cells (white arrows). (C) Confocal 20 μm z-projection of 6 dpf *Tg(gfap*:*nucGFP)* ventricle stained with glutamine synthetase. nucGFP^***+***^ cells (white arrowheads) colocalize with glutamine synthetase^***+***^ cells (white arrows). (D) Quantification of the percentage of *gfap*:*nucGFP*^***+***^ cells that express *slc1a3b*:*nls-tdTomato* (*n =* 124 cells) or GS (*n* = 76 cells). (E) Quantification of the percentage of *slc1a3b*:*nls-tdTomato*^***+***^ (*n* = 115 cells) or GS^**+**^ (*n* = 15 cells) cells that express *gfap*:*nucGFP*. (F) Confocal 10 μm z-projection of a 6-dpf *Tg(slc1a3b*:*myrGFP-P2A-H2AmCherry)*^***+***^ cell morphology. (G, H) Confocal 20 μm z-projections of (G) 6 dpf *Tg(gfap*:*nucGFP)* ventricle and (H) adult *Tg(gfap*:*nucGFP)* ventricle stained with Mef-2A. The nucGFP^***+***^ cells (white arrowhead) do not colocalize with Mef-2A puncta. Inlet area is denoted by white dotted box. (I) Quantification of percentage of *gfap*:*nucGFP*^***+***^/Mef-2A^***+***^ cells per 6 dpf (*n =* 211 cells) and adult ventricles (*n* = 49 cells). (J) Confocal 10 μm z-projection of adult *Tg(gfap*:*gfp)* ventricles stained with MBP and AcTub. MBP (white arrow) does not colocalize to *gfap*^*+*^ cells (white arrowhead). Inlet areas are denoted by white box. Data are represented as mean ± SEM. Scale bar equals 10 μm. Statistics summarized in S1 Table. See S1 Data for raw data. AcTub, acetylated tubulin; dpf, days postfertilization; GS, glutamine synthetase; MBP, myelin basic protein; V, ventricle.

In order to determine the identity of the *gfap*^*+*^ cells in the heart, we next sought to test if cardiac *gfap*^*+*^ cells labeled with other cellular markers that define specific cell types. First, we tested cell identity using transgenic lines that label the astrocyte-specific gene *glast/slc1a3b* [42], *Tg(slc1a3b*:*myrGCaMP6-P2A-H2AmCherry)*, and *Tg(slc1a3b*:*myrGFP-P2A-H2AmCherry)* [43]. These lines use regulatory regions of *slc1a3b/glast* to label the membrane with either GFP or GCaMP6 and label the cellular nuclei with mCherry [43]. We additionally used *Tg(gfap*:*nucGFP)* animals, which uses regulatory regions of *gfap* to mark cellular nuclei with GFP [44]. To test if the *gfap*^*+*^ population colocalized with *slc1a3b/glast*, we fixed *Tg(gfap*:*nucGFP);Tg(slc1a3b*:*myrGCaMP6-P2A-H2AmCherry)* animals at 6 days postfertilization (dpf) and stained with GFP to distinguish nucGFP from myrCGaMP6. In these animals, nucGFP^+^; H2AmCherry^+^ nuclei were detectable in the heart (**Fig 2A**), supporting the hypothesis that the *gfap*^*+*^ population has astroglial properties. To further confirm that this population expresses astroglial genes, *Tg(gfap*:*nucGFP)* animals were either injected with a *slc1a3b*:*nls-tdTomato* construct (**Fig 2B**) or stained with the astrocyte marker, glutamine synthetase (**Fig 2C**), and the percentage of nucGFP^+^/tdTomato^+^ and nucGFP^+^/glutamine synthetase^+^ cells per heart were scored. Given that *gfap* is expressed at different developmental time points in astrocytes as compared to *glast*/*slc1a3b* and GS [45], we would expect that only a portion of the *gfap*^*+*^ cells would be in a developmental state to coexpress either GLAST/slc1a3b or glutamine synthetase. We found that an average of 81.12% ± 5.05% and 18.91% ± 5.59% nucGFP^+^ cells expressed either tdTomato (*n =* 11 hearts, 124 cells) or glutamine synthetase (*n* = 5 hearts, 76 cells), respectively (**Figs 2D and** S1A). Conversely, an average of 86.15% ± 4.11% tdTomato^+^ cells and 93.33% ± 6.67% glutamine synthetase^+^ cells expressed nucGFP (**Fig 2E**). Together, these data support the hypothesis that a majority of *gfap*:*nucGFP*^+^ cells also express astroglial-specific markers. Next, we performed additional analysis using *Tg(gfap*:*nucGFP);Tg(slc1a3b*:*myrGFP-P2A-H2AmCherry)* animals and found extensive myrGFP^*+*^ processes that appear morphologically analogous to astroglia and strikingly different than published reports of heart populations like cardiomyocytes [46] (Fig **2F**).Together, this supports the hypothesis that the *gfap*^*+*^ cells could be an astroglial-like population.

To further test identity, we next asked if these cells colocalized with other markers of known cardiac populations. We began by asking if the *gfap*^*+*^ cells were a cardiomyocyte population and stained either 6 dpf (**Fig 2G**) or adult (**Fig 2H**) *Tg(gfap*:*nucGFP)* hearts with MEF-2A, which is expressed in the nuclei of cardiomyocytes [47]. We found that an average of 0.90% ± 0.52% and 1.80% ± 1.80% of nucGFP^*+*^ cells localized with MEF-2A at 6 dpf (*n =* 21 hearts, 211 cells) and in the adult (*n* = 5 hearts, 49 cells), respectively (**Figs 2I and** S1B). Taken together, these data with the morphological analysis of *glast*^*+*^ cells suggest that the *gfap*^*+*^ population are unlikely to be cardiomyocytes. We also could not detect costaining with myelin basic protein (MBP), which marks myelinating cells, and thus likely ruling out a myelinating Schwann cell identity (**Fig 2J**). In addition, *gfap*^*+*^ cells in the heart also did not colocalize with NG2, a marker for pericytes. Lastly, a portion of *gfap*^*+*^ cells were not positive for Vimentin (**S1C and S1D Fig**). Given that astroglia in the nervous system can be *glutamine synthetase*^*+*^, *gfap*^*+*^, *glast*^*+*^, and *Vim*^*+*^ [48], we propose that these cells in the heart could serve as an astroglia subpopulation.

**Fig 3:**
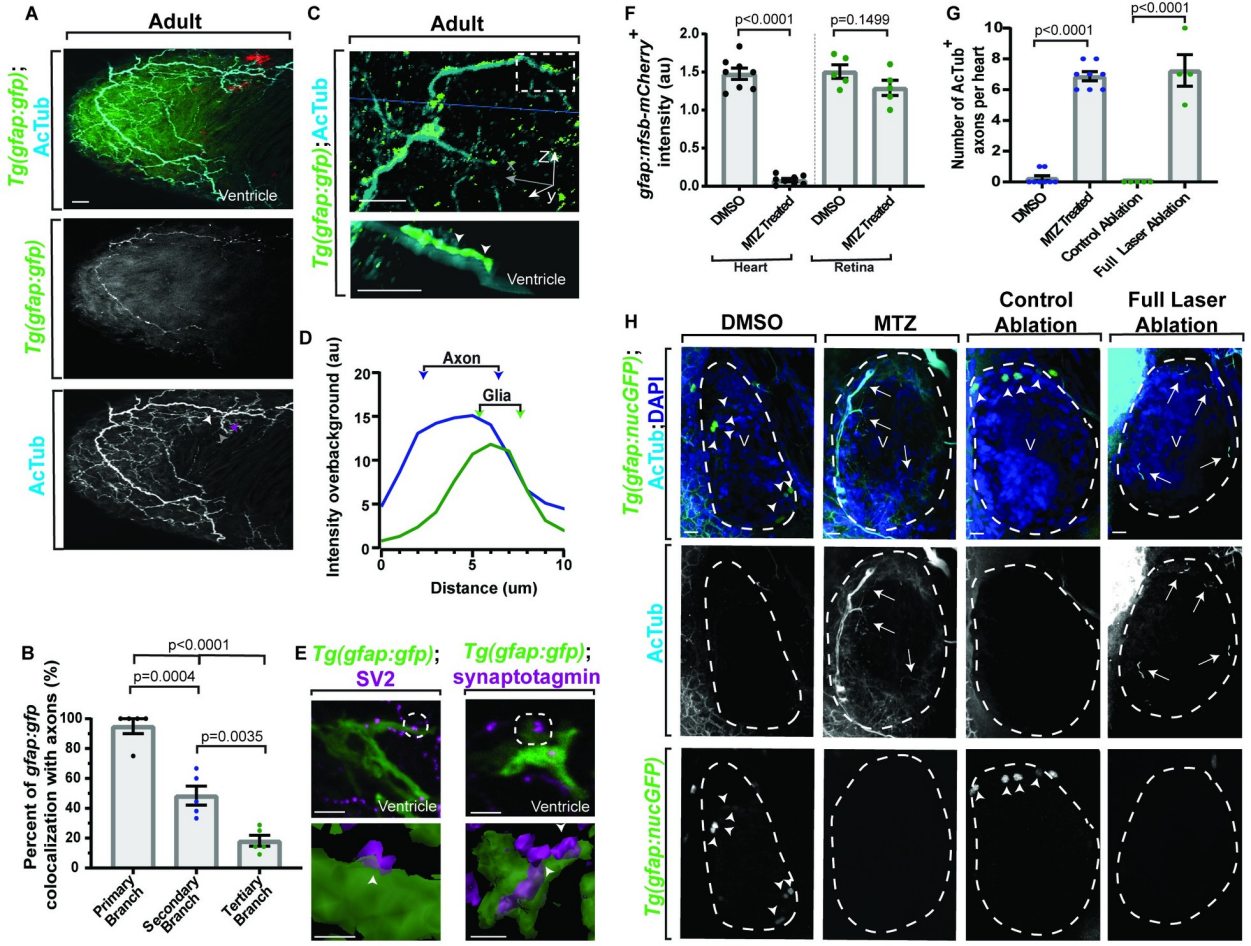
Adult *gfap*^*+*^ cells localize with neurons and synapses and impact neuronal development. (A) Confocal 10 μm z-projection from adult *Tg(gfap*:*gfp)* heart stained with AcTub. Axons have primary (white arrowhead), secondary (purple arrowhead), and tertiary (gray arrowhead) branches. (B) Quantification of colocalization between primary, secondary, and tertiary branches of axons and *gfap*^*+*^ cells. (C) Top: confocal 10 μm z-projection of adult *Tg(gfap*:*gfp)* heart stained with AcTub and rotated 90°. Bottom: 3D rendering of the AcTub and GFAP (white dotted box) are presented below. The *gfap*^*+*^ cells associate with axons (white arrowheads). (D) Representative quantification of intensity over distance of AcTub and GFAP. (E) Top: confocal maximum z-projection of adult *Tg(gfap*:*gfp)* ventricles stained with SV2 or Synaptotagmin. Bottom: 90°-rotated 3D rendering of contact points (white arrowheads) between GFAP and SV2 or Synaptotagmin. (F) Quantification of *Tg(gfap*:*nsfb-mCherry*^***+***^*)* intensity in the heart or retina (location control) after injection of DMSO or MTZ. (G) Quantification of axonal number after MTZ or full laser ablation of nucGFP^***+***^ cells. (H) Confocal maximum z-projection of ventricle (white dotted outline) of 6 dpf *Tg(gfap*:*nucGFP)* with either MTZ injection or full laser ablation of nucGFP^***+***^ cells and stained with AcTub. Arrows indicate increased innervation and arrowheads indicate nucGFP^***+***^ cells. Data are represented as mean ± SEM. Scale bar equals 10 μm. Statistics summarized in S1 Table. See S1 Data for raw data. AcTub, acetylated tubulin; dpf, days postfertilization; MTZ, metronidazole; V, ventricle.

Glial cells, like astroglia, would be expected to interact with neurons and synapses [1,2,5,49,50]. Since our data suggest a localization between these cell types (**Fig 1E**), we next used a *Tg(gfap*:*gfp)* animal and asked to what degree do the GFP^*+*^ processes localize to different AcTub^*+*^ axonal branches and quantified the percentage of GFP^*+*^ processes that had intensity overlap in the *x-y* plane with primary, secondary, and tertiary AcTub^*+*^ axons. In these areas, GFP^*+*^ processes are localized with AcTub^*+*^ axons (*n =* 5 hearts), with a decrease in localization of the lower order branches (**Fig 3A and 3B**; primary, 95.00 ± 5.00%; secondary, 48.51 ± 6.39%; tertiary, 18.29 ± 3.68%; *p* < 0.0001, one-way ANOVA). We next generated a 3D rendering of *Tg(gfap*:*gfp)* hearts stained with AcTub (**Fig 2C**), rotated the rendering 90°, and drew a line along the diameter of the axon to measure intensity of GFP and AcTub over distance (**Fig 3D**). A representative graph shows that GFP^*+*^ cells associate on top of the axon but did not fully ensheath the axon (**Fig 3D**), arguing against the possibility that they are an ensheathing Schwann cell. We next asked if the *gfap*^*+*^ population had the potential astrocyte-like [50,51] or enteric glia-like [52–54] ability to associate with synapses. Staining of *Tg(gfap*:*gfp)* hearts with the synaptic vesicle markers, SV2 (*n =* 5 hearts) and Synaptotagmin (*n* = 6 hearts) [55,56], revealed that GFP^*+*^ processes partially cover synapses in adult tissue (**Fig 3E**). Taken together, the above data are consistent with the hypothesis that this *gfap*^*+*^ population is a glial-like cell in the heart.

Astroglia in the nervous system aid in neuronal morphogenesis, and, thus, we expect if *gfap*^*+*^ cells function as glia that they would impact neuronal development [19,45]. While neurons in the CNS normally precede the development of astroglia [19], internal innervation of the zebrafish heart is absent early in development [57]. We therefore tested the possibility that *gfap*^*+*^ cells could inhibit the intraneuronal populations. Several complementary approaches were used to ablate *gfap*^*+*^ cells. First, the nitroreductase (*nsfb*/NTR)/metronidazole (MTZ) ablation system [58] was used to selectively target *gfap*^*+*^ cells in *Tg(gfap*:*nfsb-mcherry);Tg(gfap*:*nucGFP)* animals. In this technique, *Tg(gfap*:*nfsb-mcherry)* animals use regulatory regions of *gfap* to mark the cytosol with mCherry, which is tagged with NTR [59]. However, in order to visualize distinct cells, *Tg(gfap*:*nucGFP)* was also used to see individual nuclei. Therefore, cells that are tagged with NTR in *Tg(gfap*:*nfsb-mcherry)* animals will undergo cell-specific death when exposed to the drug, MTZ. While MTZ is normally provided as a global treatment, abundant *gfap*^*+*^ cells populate the zebrafish CNS early in development [44]. In order to selectively target cells of the heart as compared to a global drug treatment, MTZ was injected into the caudal vein, which transports blood to the heart, at 3 to 5 dpf to keep CNS *gfap*^*+*^ cells intact. To confirm treatment, mCherry^*+*^ intensity was scored in the heart and retina (**Fig 3F**). Intensity of mCherry in the retina was unaffected (*n =* 5 retinas, *p* = 0.1499, unpaired *t* test), while mCherry^*+*^ intensity in the heart was significantly decreased (*n =* 8 hearts, *p* < 0.0001, unpaired *t* test), indicating a successful treatment. In a second complementary approach, focal ablations with a diffraction-limited pulsed laser were used to ablate the nuclei of individual *gfap*^*+*^ cells at 5 dpf throughout the heart (*n =* 10 hearts) (**Fig 3G and 3H**). To target these cells, we used *Tg(gfap*:*nucGFP)* animals. Animals in both conditions were fixed at 6 dpf and stained with AcTub, and axon abundance was scored. Ablation of mCherry^*+*^/nucGFP^*+*^ hearts with MTZ (*n* = 8 hearts) led to an average increase from 0.25 ± 0.16 axons in DMSO animals (*n* = 8 hearts) to 6.88 ± 0.30 axons (**Fig 3G and 3H**; *p* < 0.001, unpaired *t* test). This was recapitulated in hearts that had full laser ablation of nucGFP^*+*^ (*n* = 4 hearts) cells, with an average increase from 0.00 ± 0.00 axons in the control ablation group (*n* = 5 hearts) to an average of 7.25 ± 1.03 axons (**Fig 3G and 3H**; *p* < 0.001, unpaired *t* test). These data suggest that *gfap*^*+*^ cells serve to inhibit or delay neuronal innervation early in development, in either case placing these cells central to neuronal innervation. Hereafter, we refer to these cells as cardiac nexus glia (CNG) due to their net-like pattern and astroglial properties.

### Cardiac nexus glia originate from the neural crest and develop in the heart by 4 dpf

**Fig 4:**
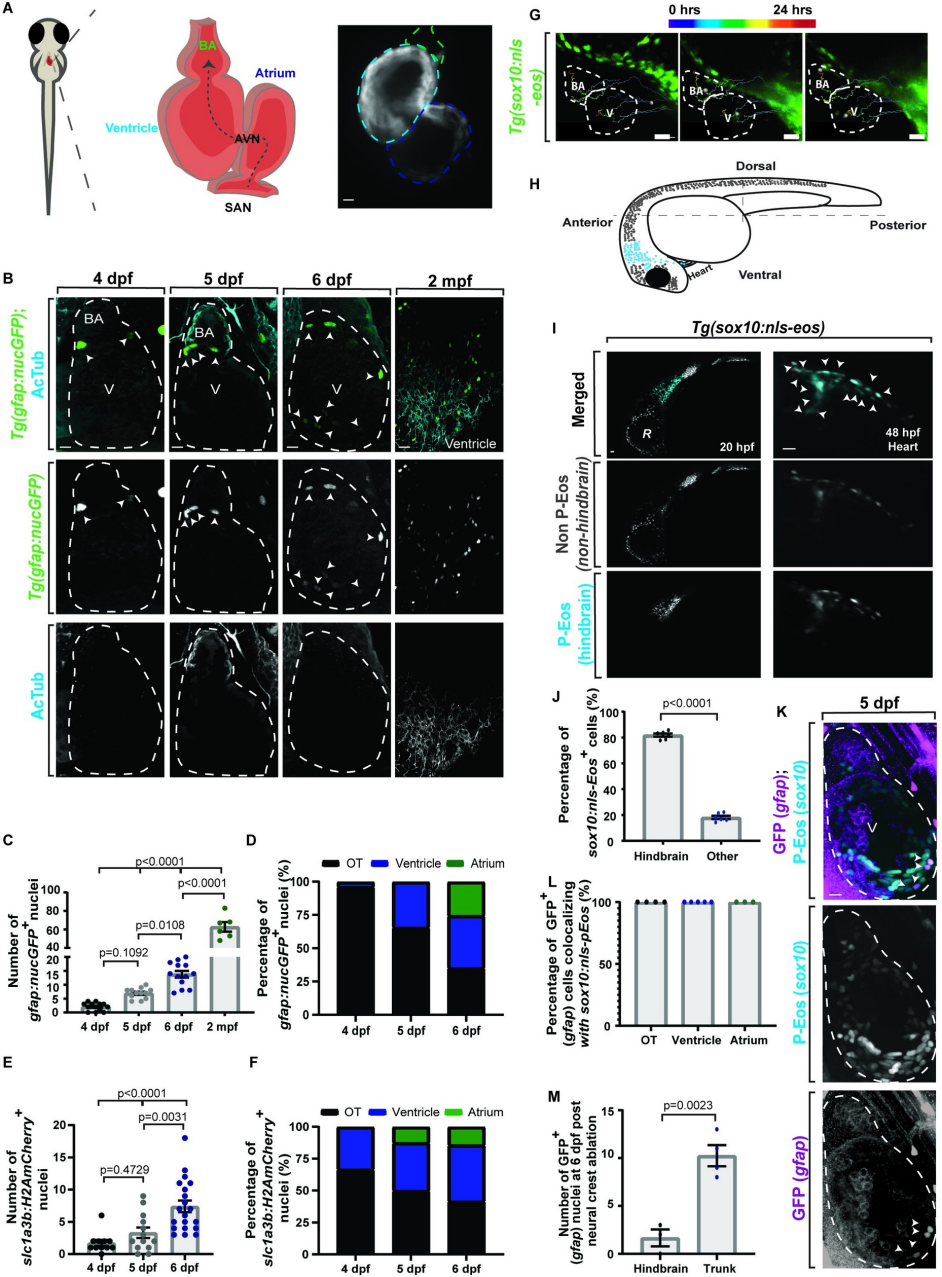
CNG originate from the neural crest and develop in the heart by 4 dpf. (A) Schematic of embryonic heart showing the BA (green), ventricle (cyan), atrium (dark blue), AVN, and SAN. Blood flows unidirectionally (gray dotted arrow). (B) Confocal maximum z-projections of heart (white dotted outline) at 4, 5, 6 dpf, and 2 mpf *Tg(gfap*:*nucGFP)* stained with AcTub. nucGFP^***+***^ cells (white arrowheads) increase over time. (C) Quantification of *Tg(gfap*:*nucGFP)*^***+***^ cell number over time. (D) Quantification of *Tg(gfap*:*nucGFP)*^***+***^ spatiotemporal location from 4–6 dpf. (E) Quantification of *Tg(slc1a3b*:*myrGFP-P2A-H2AmCherry)*^***+***^ cell number over time. (F) Quantification of *Tg(slc1a3b*:*myrGFP-P2A-H2AmCherry)*^***+***^ spatiotemporal location from 4–6 dpf. (G) Confocal maximum z-projection of a 24-hour time-lapse starting at 24 hpf in *Tg(sox10*:*nls-eos)* with migration path. Anterior neural crest cells migrate to both the outflow and inflow tract (white dotted outline). (H) Schematic of hindbrain photoconversion in *Tg(sox10*:*nls-eos)* embryos. Photoconverted cells are indicated by blue dots. (I) Confocal maximum z-projection of a 24-hour time-lapse starting at 24 hpf in *Tg(sox10*:*nls-eos)* with hindbrain neural crest photoconversion (PC). Photoconverted Eos^*+*^ cells (white arrowheads) are found throughout the heart. (J) Quantification of percentage of Eos^*+*^ cells with a hindbrain neural crest origin. (K) Confocal maximum z-projection of 5 dpf *Tg(sox10*:*nls-eos);Tg(gfap*:*nucGFP)* hearts that are photoconverted in the hindbrain at 24 hpf and stained with GFP at 5 dpf. All GFP^*+*^ cells coexpress photoconverted Eos (white arrowheads). (L) Quantification of percentage of GFP^*+*^ cells that coexpress photoconverted Eos at 5 dpf. (M) Quantification of number of GFP^*+*^ cells at 6 dpf after either hindbrain or trunk neural crest ablation. Data are represented as mean ± SEM. Scale bar equals 10 μm. Statistics summarized in S1 Table. See S1 Data for raw data. AcTub, acetylated tubulin; AVN, atrioventricular node; BA, bulbous arteriosus; CNG, cardiac nexus glia; dpf, days postfertilization; hpf, hours postfertilization; mpf, months postfertilization; OT, outflow tract; R, retina; SAN, sinoatrial node; V, ventricle.

It is currently thought that cardiomyocytes, and a further undefined cell population, are generated from the cardiac neural crest [60]. While the identity of the second group of cells is less understood, peripheral glia are commonly derived from the neural crest. Given this glial connection, we were next motivated to ask how these *gfap*^*+*^ cells develop and function. To test the development of CNG cells, we used the transparent advantage of the zebrafish embryo to visualize all chambers of the heart (**Fig 4A**). Since we can detect *gfap*^*+*^ cells at 6 dpf, we first asked when do the cells first appear in the heart and tracked nucGFP^*+*^ cells in the embryonic heart over time using *Tg(gfap*:*nucGFP)* animals (**Fig 4 B-D**). Developmental analysis revealed that nucGFP^+^ cells could first be identified at 4 dpf (*n =* 13 hearts), with an average of 2.08 ± 0.40 cells (**Fig 4B and 4C**) located at the OT (bulbous arteriosus) (**Fig 4D**). By 5 dpf (*n* = 12 hearts), nucGFP^+^ cells expanded, to an average of 6.92 ± 0.54 cells (**Fig 4B and 4C**), populating the OT and the ventricle (**Fig 4D**). By 6 dpf (*n* = 13 hearts), the average of nucGFP^+^ cells significantly increased to 13.77 ± 1.20 (**Fig 4B and 4C;** *p* = 0.0108, one-way ANOVA followed by Tukey’s post hoc test). As development of the animal progresses, so does the expansion of both abundance and location. By 2 months (*n* = 6 hearts) of age, nearly 62.83 ± 5.13 nucGFP^+^ cells (**Fig 4B and 4C**) are present throughout the heart. As a complementary approach, we also scored a similar spatiotemporal pattern of expansion with *glast*^*+*^ cells in *Tg(slc1a3b*:*myrGFP-H2A-P2AmCherry)* animals (**Fig 4E and 4F**).

We next sought to define their origin. Many peripheral glial populations are derived from the neural crest [10,61]. To explore CNG origin, we first tracked the trajectory of *sox10*^*+*^ neural crest cells from their hindbrain location to their seed destination in the heart (**Fig 4G**). To do this, we performed time-lapse imaging at 24 to 48 hours postfertilization (hpf) of *Tg(sox10*:*nls-Eos)* [62], which uses regulatory regions of *sox10* to express nuclear localized Eos. We then tracked individual cells through their migration. In these movies, Eos^*+*^ cells originated from a hindbrain location and migrated ventrally to simultaneously populate the outflow and inflow tract (**Fig 4G**). To further test if the CNGs originated from the hindbrain neural crest, we fate-mapped these Eos^*+*^ cells. To do this, we used *Tg(sox10*:*nls-eos)* again but took advantage of the Eos properties, which, when exposed to UV light, switches from green emission to red emission (**Fig 4H**). In this experiment, hindbrain-located Eos^*+*^ cells were photoconverted at 24 hpf, before neural crest migration occurred (**Fig 4I**). These animals (*n =* 6 hearts) were then imaged at 2 dpf. In the heart at 2 dpf, an average of 81.87% ± 1.27% of the *sox10*^*+*^ cells expressed photoconverted-Eos (p-Eos) (**Fig 4J**; *p* < 0.0001, unpaired *t* test). Consistent with previous reports that cardiomyocytes can be generated by neural crest, a subpopulation of these cells localized to the ventricle and atrium [63]. However, p-Eos^+^ cells were also localized to the OT, the anatomical location where *gfap*^*+*^ cells can first be detected. To then ask if these *sox10*^*+*^ cells from the neural crest generate *gfap*^*+*^ cells, we repeated the photoconversion experiment with *Tg(sox10*:*eos); Tg(gfap*:*nucGFP)* (*n =* 5 hearts) and then visualized nucGFP with an antibody against GFP to differentiate from Eos^+^ cells (**Fig 4K**). In this experiment, p-Eos will mark cells that were derived from the hindbrain neural crest, and the GFP antibody will indicate their conversion into *gfap*^*+*^ cells (**Fig 4K**). In 5 dpf hearts, 100% of the GFP^*+*^ cells were marked by p-Eos (**Fig 4L**). These data support the hypothesis that nexus glia are neural crest derived.

To further test the origin of CNGs, we next performed laser cell ablation experiments to reduce specific neural crest origins. In this paradigm, p-Eos^+^ cells in *Tg(sox10*:*eos); Tg(gfap*:*nucGFP)* were focally ablated with a diffraction-limited pulsed laser from 24 to 36 hpf during their migration from the hindbrain (*n* = 3 hearts). As a control, we used ablation of neural crest cells that originate from the trunk but do not migrate to the heart (*n* = 4). We then used an antibody to GFP to quantify *gfap*^+^ cells at 6 dpf. We found a significant reduction (**Fig 4M**; *p* = 0.0023, unpaired *t* test) in the GFP^+^ population to an average of 1.67 ± 0.88 cells in the hindbrain ablation group as compared to the trunk group, which had an average of 10.25 ± 1.11 cells. These data further indicate that CNGs are derived from the hindbrain neural crest. Collectively, these data provide experimental evidence that CNGs are neural crest derived.

### Molecular dissection of astroglia genes in the heart across species

**Fig 5:**
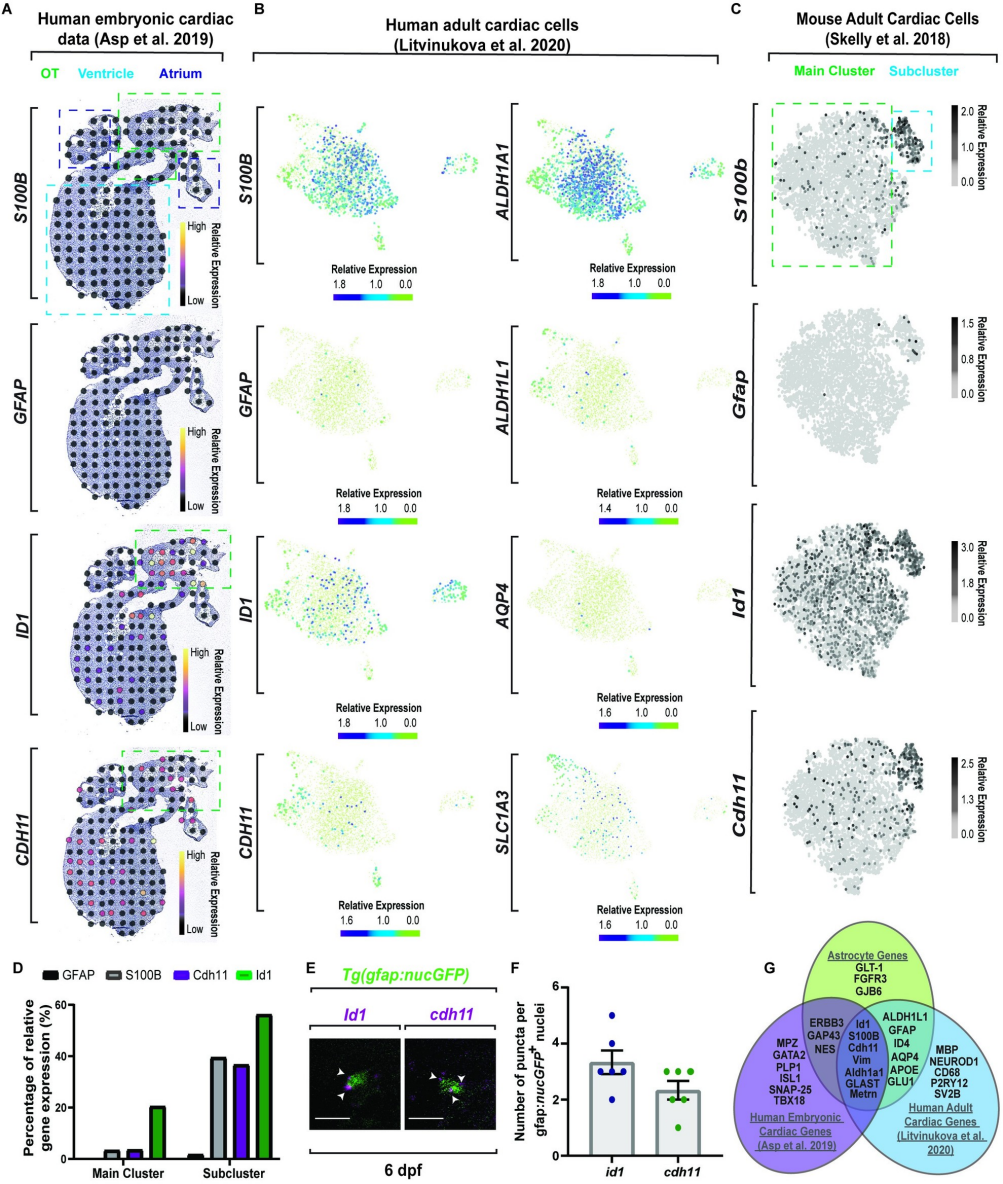
Molecular dissection of astroglia genes in the heart across species. (A) Spatiotemporal sequencing [64] of human embryonic hearts. Developmental astrocyte-associated genes are expressed. Dot color denotes relative transcript expression based on raw reads per spot. (B) Single-cell sequencing [65] from the neuronal cluster of human adult hearts. Scale bar denotes relative gene expression. (C) Single-cell sequencing [66] of astrocyte genes in the fibroblast cluster from adult mouse tissue. Scale bar denotes relative gene expression. (D) Quantification of published adult mouse single-cell sequencing [66] from the fibroblast cluster indicates a conserved subcluster with astrocyte gene up-regulation. (E) Confocal z-section of 6 dpf *Tg(gfap*:*nucGFP)* zebrafish embryos with *id1* or *cdh11* RNAScope. i*d1/cdh11*^*+*^ puncta (white arrowheads) localize to nucGFP^*+*^ cells. (F) Quantification of *id1* and *cdh11* RNAScope puncta per cell. (G) Venn diagram of published sequencing data from human embryonic hearts [64], human adult heart [65], and classic astrocyte genes. Data are represented as mean ± SEM. Scale bar equals 10 μm. Statistics summarized in S1 Table. See S1 Data for raw data. dpf, days postfertilization; OT, outflow tract.

Given their neural crest origin, we next sought to understand the molecular mechanisms of their differentiation. To first do this, we reanalyzed single-cell sequencing datasets of embryonic (**Fig 5A**) [64] and adult cardiac tissue **(Fig 5B and 5C)** [65,66], focusing on astroglia properties and potential conservation in humans. We hypothesized that astrocyte-like genes that overlapped with embryonic data would likely be early differentiation genes, whereas astrocyte-like genes that overlap with adult cardiac cells would present a more mature, differentiated cell type. Given that precursors to astrocytes normally express *VIM* and only switch gene expression to *GFAP* after differentiation [67], we also hypothesized that *GFAP* would have less expression levels particularly in the embryonic sequencing dataset. The embryonic dataset revealed limited expression of the mature astrocyte genes, *S100B* and *GFAP*, but showed expression of *ID1* and *CDH11* (**Fig 5A**). Additional analysis of the adult sequencing dataset provided a robust expression of astrocyte markers, with *S100B*, *GFAP*, *ID1*, *CDH11*, *ALDH1A1*, *AQP4*, and *SLC1A3*, and the astrocyte-specific gene *ALDH1L1* [68,69] (**Fig 5B**). Analysis of adult mouse cardiac cells [66] also corroborated an astrocyte-like cell type in mice that was enriched for *S100b*, *Id1*, and *Cdh11*, as well as limited *Gfap* expression (**Fig 5C**). Other notable astrocyte genes included *Clu*, *Vim*, *Apoe*, *Cst3*, and *Glu1* (**S2 Data**). This enrichment appeared localized together in a subcluster away from the main cluster of fibroblasts in the dataset (**Fig 5D**). Collectively, these data support the possibility that an astrocyte-like population seeds the vertebrate heart.

**Fig 6:**
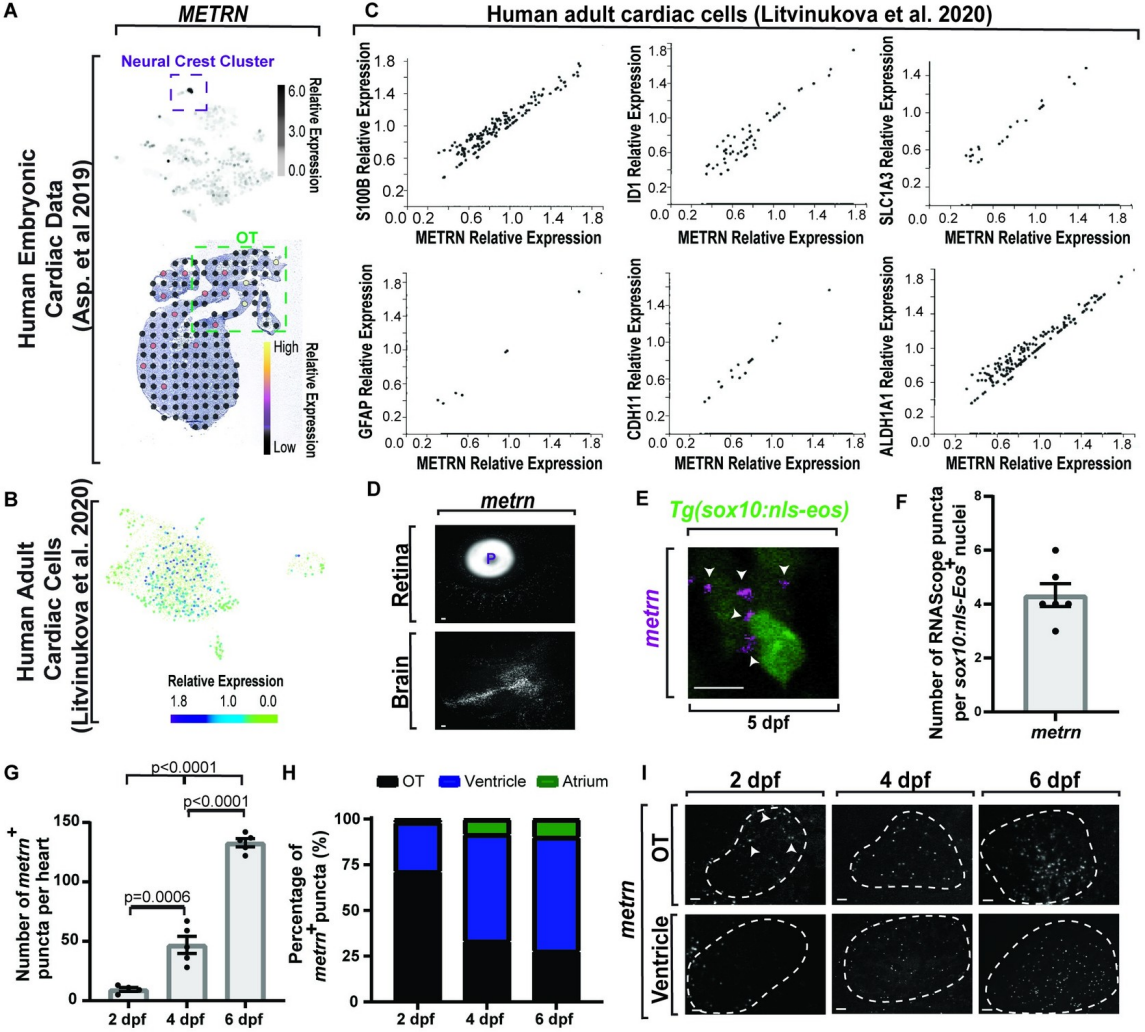
Meteorin coexpresses with astroglial genes and is expressed in the heart across species. (A) Top: single-cell sequencing of *METRN* in human embryonic hearts [64]. *METRN* is most up-regulated in the neural crest cluster (purple dotted box). Scale bar denotes relative gene expression. Bottom: spatiotemporal sequencing [64] of *METRN* in human embryonic hearts. Dot color denotes relative transcript expression. (B) Single-cell sequencing [65] from the neuronal cluster of human adult hearts. Scale bar denotes relative gene expression. (C) Coexpression of *METRN* and astroglial genes from human adult single-cell sequencing data [65]. Axes denote relative gene expression. (D) Confocal 20 μm z-projection of 6 dpf zebrafish animals with *metrn* RNAScope. *Metrn* localizes to the retina and the brain. (E) Confocal z-sections of *metrn* RNAScope 5 dpf *Tg(sox10*:*nls-eos)* zebrafish embryos. *Metrn*^*+*^ puncta (white arrowhead) localize to Eos^*+*^ cells. (F) Quantification of *metrn*^*+*^ puncta per cell in the heart. (G) Quantification of the number of *metrn* mRNA puncta in the heart at 2, 4, and 6 dpf. (H) Quantification of locational development of *metrn* mRNA puncta in the heart at 2, 4, and 6 dpf. (I) Confocal z-sections of *metrn* mRNA puncta (white arrowheads) across location and time in the heart (white dotted outline). Data are represented as mean ± SEM. Scale bar equals 10 μm. Statistics summarized in S1 Table. See S1 Data for raw data. dpf, days postfertilization; OT, outflow tract; P, pupil.

To complement this mouse and human analysis in zebrafish, we used RNAscope on zebrafish cardiac tissue to detect expression of the genes that were identified as potential molecular markers of the glial-like subcluster. On average, 2.33 ± 0.33 *cdh11*^*+*^ puncta (*n* = 6 hearts) and 3.33 ± 0.42 *id1*^*+*^ puncta (*n* = 6 hearts) localized with *gfap*^*+*^ cells (**Fig 5E and 5F**). These results are consistent with the hypothesis that a conserved cell population with glial-like properties exists within the heart. To shorten the list of candidate determinants of CNGs, we used the overlap of human embryonic and adult cardiac transcripts and astrocyte-like genes (**Fig 5G**). In this overlap, we identified *METRN*, which is required for GFAP/Gfap/*gfap*^*+*^ astrocytes to differentiate [70,71]. In the developmental dataset, we found that sections of embryonic human hearts displayed expression of *METRN* localized to the OT (**Fig 6A**), the area where *gfap*^*+*^ cells first seed the zebrafish heart (**Fig 4D**). We also found that scRNA sequencing of human embryonic heart cells showed that *METRN* expression is enriched, specifically in cells that cluster as neural crest [64] (**Fig 6A**). The human adult sequencing data displayed abundant *METRN* expression in the “neuronal” cell cluster (**Fig 6B**), which is coexpressed in single cells with the glial-associated gene, *S100B*, as well as *GFAP*, *ID1*, *CDH11*, *GLAST/SLC1A3*, and *ALDH1A1* (**Fig 6C**). Together, these data are consistent with the hypothesis that *METRN*^*+*^ neural crest–derived cells seed the OT humans.

To test the possibility that *metrn*^*+*^ neural crest cells seed the OT of zebrafish, we assayed *metrn* expression in embryonic zebrafish heart with RNAscope *in situ* hybridization. To first confirm the specificity of *metrn* RNAScope, we assayed the retina and brain with the *metrn* probe in zebrafish, which has been shown to express *metrn* [71] (**Fig 6D**). To next determine if *metrn* was expressed during cardiac glial development, hearts from *Tg(sox10*:*nls-Eos)* animals (*n =* 5 hearts) were assayed with the *metrn* RNAscope probe (**Fig 6E**). At 4 dpf, Eos^*+*^ cells expressed an average of 4.33 ± 0.42 *metrn*^*+*^ puncta per cell (**Fig 6E and 6F**). We further identified an increase in the number of *metrn* puncta beginning at 4 dpf and continuing to 6 dpf (**Fig 6G**), which aligns with when we first can detect CNGs in the heart (**Fig 4B and 4C**). In addition, *metrn* puncta display in a similar spatiotemporal pattern as *gfap*^*+*^ and *glast*^*+*^ cells from the OT to the inflow tract (**Fig 6H and 6I**). Together, these data are consistent with the idea that *metrn* is spatiotemporally localized in an area to potentially impact CNG differentiation.

### Meteorin acts as a molecular determinant of cardiac nexus glia through Jak/Stat3 signaling

**Fig 7:**
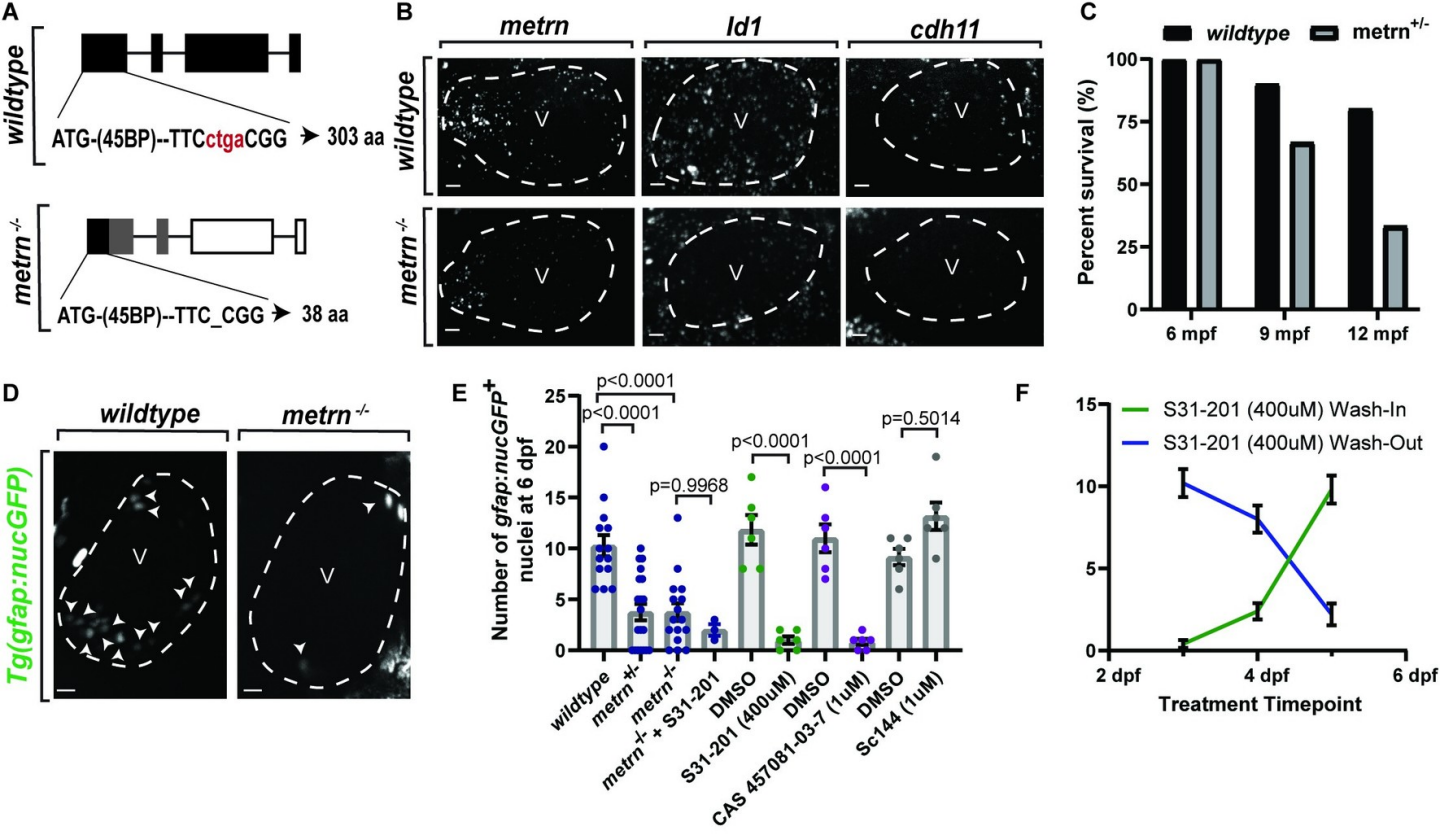
Meteorin acts as a molecular determinant of CNG. (A) Schematic representation of *metrn* CRISPR mutation (red). (B) Confocal z-sections of *metrn*, *id1*, and *cdh11* mRNA in the ventricle (white dotted outline) at 6 dpf in wild-type and *metrn*^*−/−*^ embryos. (C) Quantification of percent survival of adult wild-type and *metrn*^*+/−*^ animals. (D) Confocal maximum z-projection of nucGFP^*+*^ number (white arrowheads) in the ventricle (white dotted outline) at 6 dpf in *Tg(gfap*:*nucGFP)* wild-type and *metrn*^*−/−*^ embryos. (E) Quantification of *Tg(gfap*:*nucGFP)*^*+*^ number at 6 dpf over various manipulations of the differentiation pathway. (F) Quantification of *Tg(gfap*:*nucGFP)*^*+*^ number after wash-in/wash-out of S31-201 at different time points. Crossover between the curves indicates a functional role between 4–5 dpf. Data are represented as mean ± SEM. Scale bar equals 10 μm. Statistics summarized in S1 Table. See S1 Data for raw data. aa, amino acid; CNG, cardiac nexus glia; dpf, days postfertilization; mpf, months postfertilization; V, ventricle.

We next asked if *metrn* is a determinant of CNGs. Using CRISPR/Cas9, we generated a mutant with a 4-bp deletion that putatively truncates the Metrn protein to 38 amino acids versus 303 amino acids in wild-type animals (**Fig 7A**). We validated this mutant by assaying mRNA with the *metrn* RNAscope probe, which targets the translated region of exon 1 and observed a decrease in mRNA puncta in *metrn*^*−/−*^ animals (*n =* 3) (**Fig 7B**). While 100.00% of wild-type (*n* = 10 animals) and heterozygous animals (*n* = 6 animals) survived to early adulthood, we noted a decrease to 33.33% survival by 12 months postfertilization (mpf) in heterozygous siblings, while wild-type animals had an 80.00% survival (**Fig 7C**). We did not detect adult homozygous siblings from that experiment. However, in other crosses that were grown to adulthood, homozygous animals could be identified (*n* = 5 out of 36 animals, 14%, chi-squared test to mendelian ratios, *p* = 0.2946).

We next scored the abundance of CNG in wild-type, *metrn*^*+/−*^, and *metrn*^*−/−*^ animals at 6 dpf in *Tg(gfap*:*nucGFP)*. *metrn*^*+/−*^ (*n =* 20 hearts) and *metrn*^*−/−*^ (*n* = 16 hearts) animals displayed overall decreased number of nucGFP^*+*^ cells in the heart from an average of 10.29 ± 1.05 cells in the wild-type group (*n* = 14 hearts) to an average of 3.75 ± 0.80 (*p* < 0.0001, one-way ANOVA with Tukey’s post hoc test) and 3.75 ± 0.86 nucGFP^*+*^ cells (*p* < 0.0001, one-way ANOVA with Tukey’s post hoc test), respectively (**Fig 7D and 7E**). To further confirm that CNGs were disrupted and not just down-regulating *gfap*, *metrn*^*−/−*^ animals also displayed less *cdh11* and *id1* (**Fig 7B**), consistent with the hypothesis that *metrn* impacts CNG.

In astrocytes, *metrn* has been reported to function through the Jak/Stat3 pathway [70]. To test in vivo if *metrn* signaling pathway impacts CNG differentiation, we treated *Tg(gfap*:*nucGFP)* animals with either a Stat3 (S31-201) or Jak1 (CAS-457061-03-7) inhibitor and compared the abundance of nucGFP^*+*^ cells to DMSO-treated animals. Such treatments were done at 4 dpf, to avoid disrupting Stat3 or Jak1 during early heart development [72]. Both Stat3 (*n =* 6 hearts) and Jak1 (*n =* 6 hearts) inhibition reduced the abundance of nucGFP^*+*^ cells to an average of 1.00 ± 0.37 (DMSO = 11.83, *n* = 6, *p* < 0.0001, one-way ANOVA followed by Tukey’s post hoc test) and 0.83 ± 0.31 (DMSO = 11.00, *n* = 6, *p* < 0.0001, one-way ANOVA followed by Tukey’s post hoc test) cells (**Fig 7E**). However, Jak/Stat3 signaling is not activated through recruitment of its receptor, *gp130*, as previously found [70] (**Fig 7E**; DMSO = 9.17 ± 0.79 cells, *n* = 6, Sc144 = 13.17 ± 1.35 cells, *n* = 6 hearts, *p* = 0.5015, one-way ANOVA followed by Tukey’s post hoc test). *metrn*^*−/−*^ animals treated with the Stat3 inhibitor (*n* = 3 hearts) also displayed a decrease in nucGFP^*+*^ cells to an average of 2.00 ± 0.58 cells, comparable to untreated *metrn*^*−/−*^ animals (**Fig 7E**; *p* = 0.9968, one-way ANOVA followed by Tukey’s post hoc test), suggesting that Stat3 functions in the same genetic pathway as *metrn* to impact CNG differentiation.

To further probe this molecular mechanism, we tested the temporal requirement of Jak/Stat3 signaling. Our data suggest that *sox10*^*+*^ neural crest cells seed the OT by 48 hpf then differentiate into *gfap*^*+*^ cells by 6 dpf (**Fig 4I and 4J**). If *metrn* signaling drives differentiation of CNG, then it should also be required from 3 to 6 dpf in zebrafish when we visualize seeding of *gfap*^*+*^ cells. To test this, we performed a “wash-in” drug treatment experiment, in which the drug is introduced to the egg water at time points before, during, or after the signaling is potentially necessary for the differentiation of a cell. Conversely a “wash-out” was also performed in which the drug is introduced prior to the differentiation of a cell and is replaced with normal egg water either during or after the necessary signaling. The number of cells is then scored and plotted for both the “wash-in/wash-out” treatments at each time point, and the overlap of the curves indicates the time that the signaling is most likely required. For this experiment, the Stat3 inhibitor was either washed into the egg water at 3, 4, or 5 dpf, or treated at 3 dpf and washed out at 4 or 5 dpf. The abundance of nucGFP^*+*^ cells was scored at 6 dpf. The overlap of these 2 curves occurred at 4.5 dpf (wash-in: *n =* 5 hearts, wash-out: *n* = 5 hearts), indicating that Stat3 signaling likely occurs at 4.5 dpf (**Fig 7F**). This developmental timing is in line with when we first can detect *gfap*^*+*^ cells that have transitioned from *sox10*^*+*^ cells.

### Cardiac nexus glia regulate heart function in a location-dependent manner

**Fig 8:**
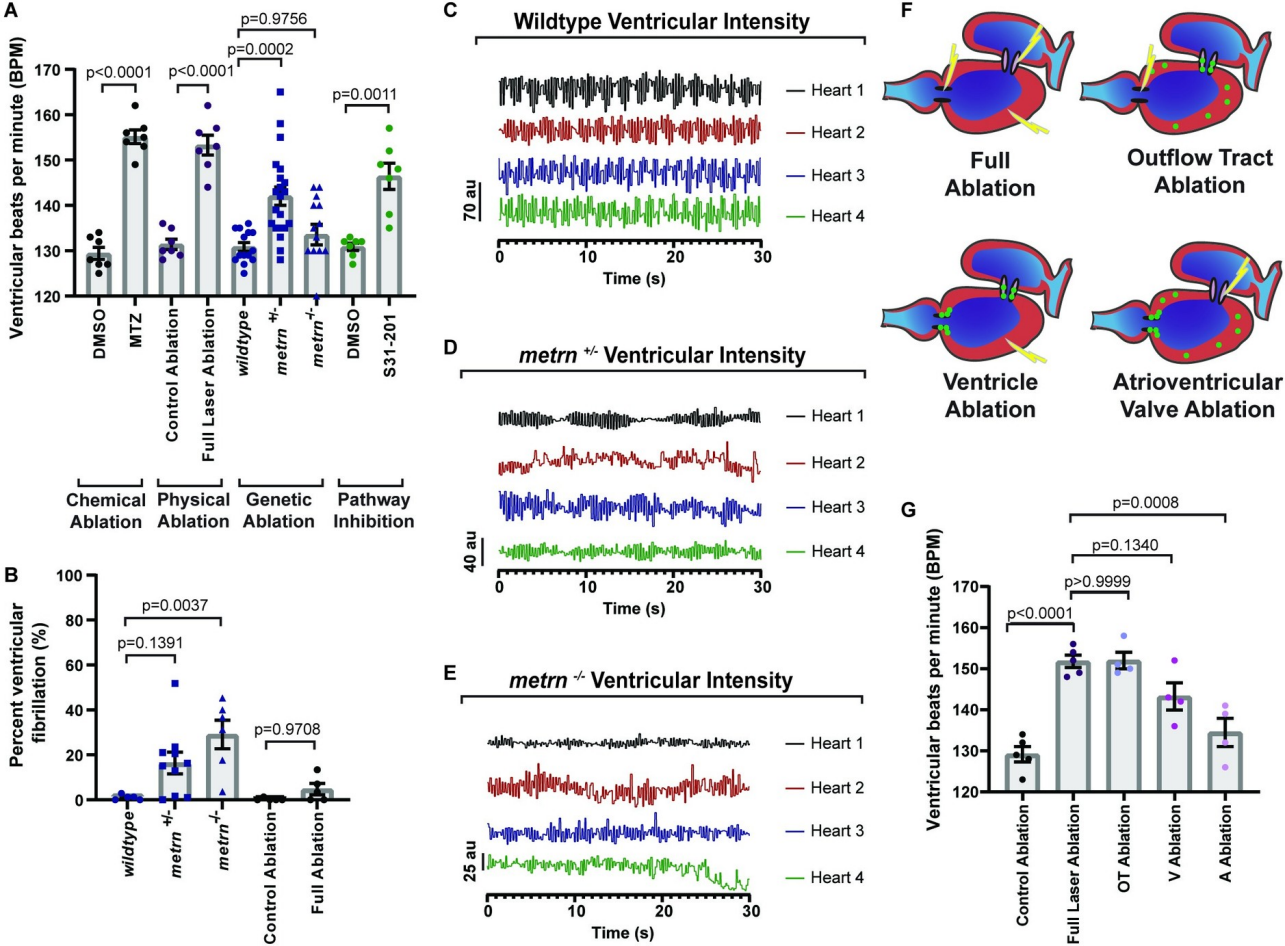
CNG regulate heart rate and rhythm. (A) Quantification of the number of ventricular contractions (bpm) at 6 dpf after various *gfap*^*+*^ ablation techniques. (B) Quantification of percent duration of ventricular fibrillation in *metrn*^*−/−*^ animals and *Tg(gfap*:*nucGFP)* animals with full laser ablation of nucGFP^*+*^ cells. (C) Representative graphs of ventricular blood flow intensity (au) over time in wild-type animals. (D) Representative graphs of ventricular blood flow intensity (au) over time in *metrn*^*+/−*^ animals. (E) Representative graphs of ventricular blood flow intensity (au) over time in *metrn*^*−/−*^ animals. (F) Schematic of *gfap*^*+*^ ablation by location. Green dots denote *gfap*^*+*^ cells. Lightning denotes ablation. (G) Quantification of number of ventricular contractions at 6 dpf after ablation of *gfap*^*+*^ by location. Data are represented as mean ± SEM. Statistics summarized in S1 Table. See S1 Data for raw data. A, atrium; bpm, beats per minute; CNG, cardiac nexus glia; dpf, days postfertilization; MTZ, metronidazole; OT, outflow tract; V, ventricle.

With origins and determinants identified, we next investigated the physiological function of CNGs. Cardiac function requires precise, unidirectional flow of electrical activity for proper contraction and relaxation of the heart chambers [16,18,73] (**S2A Fig**). In order to determine if there are any physical changes to gross morphology of the heart that would affect cardiac function, *metrn*^*−/−*^ animals were used to examine morphology in the absence of CNGs. We therefore used the shape descriptor features on ImageJ to score gross heart morphology per chamber between wild-type (*n =* 5 hearts) and *metrn*^*−/−*^ (*n* = 5 hearts) animals and found no significant changes between groups (**S2B–S2D Fig**). Given that no gross structural abnormalities could be detected to impact heart physiology, we next sought to test the role of CNGs in cardiac function by removing the *gfap*^*+*^ cells with several complementary approaches. We used wild-type (*n* = 14 hearts) (**S1 Movie**), *metrn*^*+/−*^ (*n* = 21 hearts) (**S2 Movie**), and *metrn*^*−/−*^ (*n* = 14 hearts) (**S3 Movie**) animals to examine a genetic mutation that alters CNGs. We also inhibited the differentiation pathway of CNGs with a S31-201 treatment at 4 to 5 dpf (*n* = 7 hearts). To account for the possibility that *metrn*^*−/−*^ and S31-201 treated animals may impact additional cell types, we also performed cell-specific ablation with either the cell-specific cytotoxic NTR/MTZ method of *gfap*^*+*^ cell ablation or *gfap*^+^ cell-specific focal laser ablation across the heart (**Fig 2J and 2K**). Heart rates were then quantified by scoring the number of full ventricular contractions over a 1-minute time period at 6 dpf, noted in the figures as beats per minute (bpm) (**Fig 8A**). Results showed that MTZ (*n* = 7 hearts) treated animals had an averaged increased heart rate from 129.40 ± 1.33 bpm in the DMSO group (*n* = 7 hearts) to 155.10 ± 1.50 bpm (**Fig 8A**; *p* < 0.0001, one-way ANOVA with Tukey’s post hoc test). This was recapitulated in the laser-ablated animals (*n* = 7 hearts), which had an increase from an average of 131.40 ± 1.15 bpm in the control ablation group (*n* = 7 hearts) to 153.3 ± 2.20 bpm (**Fig 8A**; *p* < 0.0001, one-way ANOVA with Tukey’s post hoc test). While *metrn*^*+/−*^ animals (*n* = 21 hearts) had an average increased heart rate of 142.00 ± 2.03 bpm (*p* = 0002, one-way ANOVA with Tukey’s post hoc test), *metrn*^*−/−*^ siblings (*n* = 14 hearts) displayed no difference in bpm as compared to wild-type animals (*n* = 14 hearts) (**Fig 8A**; wild-type = 130.90 ± 0.94 bpm, *metrn*^*−/−*^ = 133.58 ± 2.25 bpm, *p* = 0.9756, one-way ANOVA with Tukey’s post hoc test). However, disrupting the genetic pathway with S31-201 (*n =* 7 hearts) led to an increase in heart rate from an average of 130.90 ± 0.80 bpm in DMSO animals (*n* = 7 hearts) to 146.40 ± 2.90 bpm (**Fig 8A**; *p* = 0.0011, one-way ANOVA with Tukey’s post hoc test). Taken together, removal of the CNG population led to a rhythmic increase in heart rate, which is a state that is commonly referred to as ventricular tachycardia.

Severe forms of ventricular tachycardia can deteriorate into ventricular fibrillation [74], in which increased activity of the sympathetic nervous system alters either the repolarization or depolarization phase of cardiac action potentials [73]. This causes a rare but life-threatening state in which the ventricles quiver and retain blood instead of fully contracting. Although *metrn*^*−/−*^ animals did not have significantly different bpm change, arrhythmic beating of the heart was detected (**S3 Movie**). Given this observation, we next asked if heart rate variability in *metrn* mutants is due to a deterioration into ventricular fibrillation. To address this, we drew a line across the diameter of the ventricle and measured the intensity over 1 minute to quantify the blood flow through the ventricular chamber over time. Higher values indicate when blood is exiting the ventricle during contraction, and lower values indicate when blood is filling the ventricle during relaxation, and intervals between intensity change indicate if fibrillation is occurring. On average, *metrn*^*−/−*^ (*n =* 6 hearts) animals displayed ventricular fibrillation 29.07 ± 6.39% of the time, while wild-type (*n* = 5 hearts) animals only had an average of 1.08 ± 0.53% fibrillation (**Fig 8 B-E**, **S2 and S3 Movies**; *p* = 0.0037, one-way ANOVA with Tukey’s post hoc test), suggesting that the genetic mutation that alters CNGs can manifest a ventricular fibrillation phenotype. To further test the role of CNG in heart rhythm, we next used 5 dpf *Tg(gfap*:*nucGFP)* animals and focally ablated nucGFP^+^ cells with the laser and measured ventricular fibrillation at 6 dpf. In laser-ablated animals, fibrillation occurred 4.77 ± 2.53% of the time, while we could only detect fibrillation in wild-type animals 0.26 ± 0.26% of the time (**Fig 8B**; *p* = 0.9708, one-way ANOVA with Tukey’s post hoc test). However, fibrillation in laser-ablated animals was not enough to lower the tachycardia phenotype as seen in *metrn*^*−/−*^ animals (**Fig 8A**). Collectively, these data are consistent with the hypothesis that CNGs function to regulate heart rate during development and that genetic mutations that reduce CNGs can result in rhythm defects.

Given the finding that CNG cells can modulate cardiac function, we next sought to elucidate where the cells are primarily functioning. In the absence of structural abnormalities **(S2B-S2D Fig)**, ventricular tachycardia is caused by the imbalance of ionic activity in the OT [75,76]. This sporadic electrical activity can then lead to ventricular tachycardia due to changes in cardiomyocyte signaling [74,77]. Since *metrn*^*+*^ neural crest cells seed the OT in zebrafish and humans, and due to the prominence of OT dysfunction in CHD [78], we next examined if the anatomical location of CNGs differentially have an impact on heart rate. To test this, we used *Tg(gfap*:*nucGFP)* animals and measured heart rate at 6 dpf after focal laser ablation of nucGFP^*+*^ cells in either the OT (**S4 Movie**), ventricle (**S5 Movie**), atrium (**S6 Movie**), or throughout the entire heart at 5 dpf (**Fig 8F**). Full ablation (*n =* 5 hearts) of nucGFP^*+*^ cells induced an increase in heart rate to an average of 151.80 ± 1.50 bpm. This was comparable to heart rate with ablation of OT (*n* = 4 hearts) and ventricular CNGs (*n* = 4 hearts), which had an increased heart rate to an average of 152.00 ± 2.04 bpm (*p* > 0.9999, one-way ANOVA with Tukey’s post hoc test) and 143.30 ± 3.30 bpm (*p* = 0.1340, one way ANOVA with Tukey’s post hoc test), respectively (**Fig 8G**). However, heart rate was not increased with ablation of atrial CNGs (**Fig 8G**; 134.50 ± 3.43 bpm, *n =* 4, *p* = 0.0008, one-way ANOVA with Tukey’s post hoc test). These data are consistent with the hypothesis that CNGs regulate heart rate in a location-dependent manner in which OT and ventricular cells are critical for modulating embryonic heart rate.

### Cardiac nexus glia regulate heart rhythm and control cardiac function through sympathetic/parasympathetic modulation

**Fig 9:**
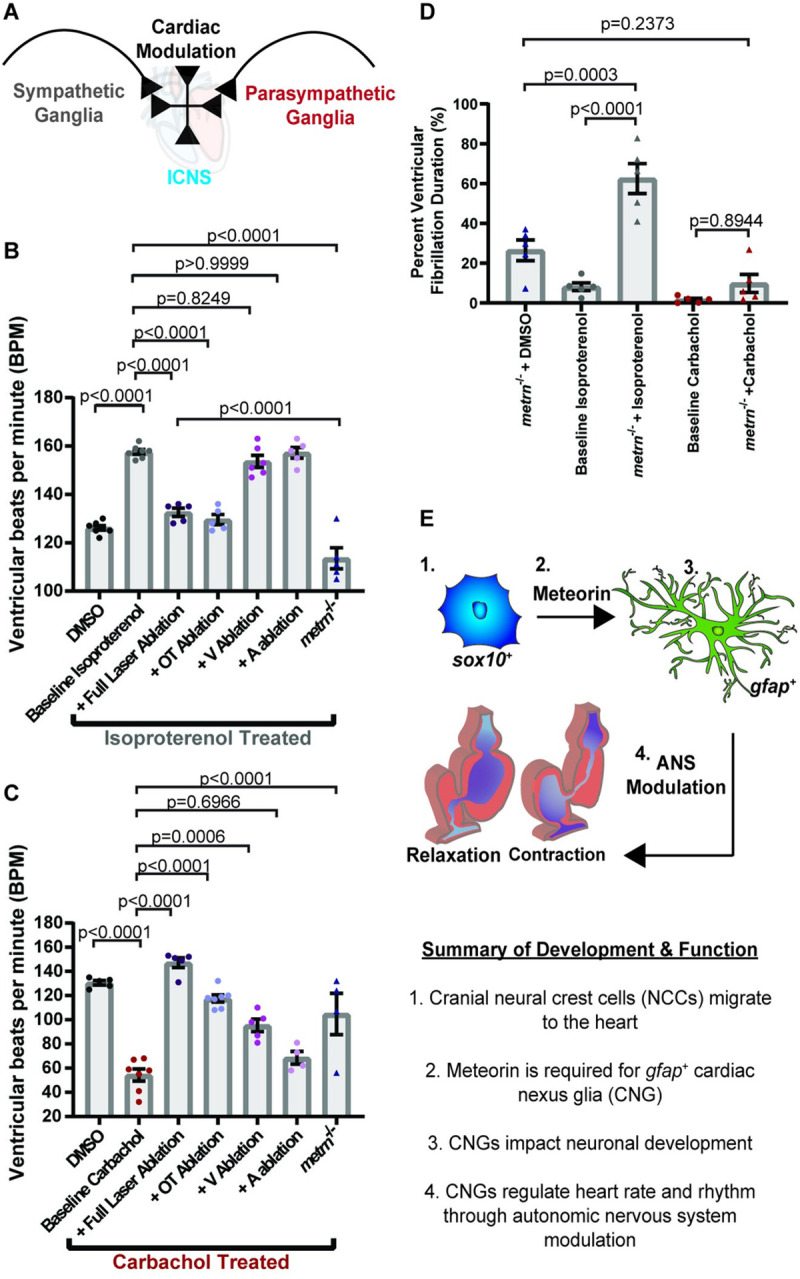
CNG control heart function through sympathetic/parasympathetic modulation. (A) Schematic of sympathetic and parasympathetic regulation of cardiac function through communication with the ICNS. (B) Quantification of the number of ventricular contractions with sympathetic agonist (isoproterenol) treatment over various location ablation of nucGFP^*+*^ cells. (C) Quantification of the number of ventricular contractions with parasympathetic agonist (carbachol) treatment over various location ablation of nucGFP^*+*^ cells. (D) Quantification of percent duration of ventricular fibrillation in *metrn*^*−/−*^ animals after treatment with the isoproterenol or carbachol. (E) Schematic of overall findings of CNG showing that they originate from the neural crest and require *metrn* to generate *gfap*^*+*^ cells that control heart rate and rhythm. Data are represented as mean ± SEM. Statistics summarized in S1 Table. See S1 Data for raw data. A, atrium; ANS, autonomic nervous system; bpm, beats per minute; CNG, cardiac nexus glia; ICNS, intracardiac nervous system; OT, outflow tract; V, ventricle.

Cardiac function is tightly regulated by a hierarchy of neuronal control, with the ICNS locally coordinating afferent and efferent signals from sympathetic and parasympathetic neurons to anatomically regulate electrical activation, propagation, and conduction (**Fig 9A**) [79]. Therefore, we next addressed if CNG cells are required for components of the parasympathetic (PSNS) or sympathetic (SNS) nervous systems. To test this, we used either *metrn*^*−/−*^ animals or we performed focal laser ablation in *Tg(gfap*:*nucGFP)* animals to fully ablate nucGFP^*+*^ cells in the heart at 6 dpf and immediately treated with the sympathetic agonist, isoproterenol, or the parasympathetic agonist, carbachol [57]. We then recorded heart rate at 1 hour posttreatment (**Fig 9B and 9C**). In nonablated animals (*n =* 7 hearts), treatment of isoproterenol predictably increased heart rate to an average of 157.60 ± 1.00 bpm as compared to DMSO-treated animals (*n* = 7 hearts, 126.10 ± 0.96, *p* < 0.0001, one-way ANOVA with Tukey’s post hoc test). However *metrn*^*−/−*^ animals (*n* = 5 hearts) and (*n* = 5 hearts) ablation of nucGFP^*+*^ cells with a laser abolished the increased heart rate from isoproterenol and had an average heart rate of 113.60 ± 4.37 (*p* < 0.0001, one-way ANOVA with Tukey’s post hoc test) and 132.60 ± 1.69 bpm (*p* < 0.0001, one-way ANOVA with Tukey’s post hoc test). This suggests that CNGs function in the sympathetic response (**Fig 9B**). In nonablated animals (*n* = 7 hearts), carbachol treatment demonstrated reduced heart rate to an average of 54.29 ± 5.02 bpm as compared to animals treated with DMSO (*n* = 5 hearts, 130.60 ± 1.81 bpm, *p* < 0.0001, one-way ANOVA with Tukey’s post hoc test). Again, both *metrn*^*−/−*^ animals (*n =* 4 hearts) and full ablation of nucGFP^*+*^ cells (*n* = 5 hearts) abolished the response to carbachol to an average heart rate of 104.80 ± 17.07 bpm (*p* < 0.0001, one-way ANOVA with Tukey’s post hoc test) and 147.20 ± 4.10 bpm (*p* < 0.0001, one-way ANOVA with Tukey’s post hoc test), indicating that the CNGs additionally function in the parasympathetic response (**Fig 9C**). Given that both OT and ventricular CNGs regulate heart rate, we next asked if CNGs function in a location-dependent manner in the ANS response (**Fig 9B and 9C**). Ablation of OT nucGFP^*+*^ cells abolished the impact of both isoproterenol (*n =* 5 hearts) to an average heart rate of 129.60 ± 2.11 bpm (**Fig 9B**; *p* < 0.0001, one-way ANOVA with Tukey’s post hoc test) and carbachol (*n* = 7 hearts) to an average heart rate of 117.60 ± 3.02 bpm (**Fig 9C**; *p* < 0.0001, one-way ANOVA with Tukey’s post hoc test). In the carbachol-treated group, ablation of ventricular nucGFP^*+*^ cells also rescued heart rate to an average of 95.40 ± 5.14 bpm (*p* = 0.0006, one-way ANOVA with Tukey’s post hoc test). Collectively, these data indicate that primarily OT CNGs regulate heart rate through regulation of the ANS.

The ANS has also been implicated in ventricular fibrillation, in which there is an overactivation of the sympathetic response [73]. While *metrn*^*−/−*^ animals treated with isoproterenol reduced the increase in heart rate, it was a more severe reduction than in the full laser ablation condition (**Fig 9B**). Given the prominence of ventricular fibrillation in *metrn*^*−/−*^ animals, we next hypothesized that sympathetic activation increases a fibrillation phenotype. Therefore, we last asked if CNGs regulate rhythm through modulation of the sympathetic and parasympathetic systems by treating *metrn*^*−/−*^ animals with isoproterenol (*n* = 5 hearts) or carbachol (*n* = 5 hearts) (**Fig 9D**). As hypothesized, increased sympathetic activity with isoproterenol led to an average increase of 62.55 ± 7.50% duration of fibrillation as compared to *metrn*^*−/−*^ animals (*n* = 5 hearts) treated with DMSO, which had an average duration of 26.50 ± 5.25% (**Fig 9D;** *p* = 0.0003, one-way ANOVA with Tukey’s post hoc test). Treatment with the parasympathetic agonist carbachol did not have a significant effect and displayed an average duration of 9.84 ± 4.56% (**Fig 9D**; *p* = 0.2373, one-way ANOVA with Tukey’s post hoc test), which is in line with the pathology of ventricular fibrillation [73]. Taken together, these data support the hypothesis that CNGs regulate rhythm through differential activity of the ANS. Our data confirm the existence of glia in the heart while providing an important role in cardiac function during development.

## Discussion

Here, we identify a glial population within the heart that supports cardiac function (**Fig 9E**). This population, like other peripheral glia, originates from the neural crest and migrates to the inflow and outflow tracts of the heart. By 3 dpf, Meteorin activates JAK/Stat3 signaling to induce *gfap*^*+*^ cellular differentiation. Analysis of human embryonic and mouse adult sequencing data suggests an up-regulation of astroglial genes, and RNAscope of zebrafish embryos demonstrates an astrocyte-like molecular identity in CNG. We further show that this *gfap*^*+*^ cell population is conserved across zebrafish, mice, and humans. Similar to Burkhard and Bakkers (2018), we found no neuronal innervation of the heart this early in development in wild-type zebrafish animals [57]. Contrarily, there was minimal but increased axonal innervation when CNG were ablated, indicating that CNG inhibit or delay cardiac axonal innervation. Furthermore, we have demonstrated that *gfap*^*+*^ cells primarily in the OT work with sympathetic and parasympathetic ganglionic inputs to inhibit ventricular tachycardia and fibrillation. These data thus indicate an integral role of CNG in regulating heart function.

Currently, identification of astroglia in organs has lagged behind CNS astroglia research. Here, we show in vivo that CNG display distinguishing features expected of an astroglial cell [19,51,80]. They express astroglial-associated genes, including *gfap*, *glast*, glutamine synthetase, and *id1*, and associate with neurons and synapses. They further impact neuronal development and are dependent on an astroglial determinant, Meteorin [70]. To date, most research on Meteorin has been performed in vitro and has demonstrated a key role in both differentiation of astrocytes, Bergmann glia, and Müller glia in the CNS as well as regulation of neurite extension [70,71]. While secretion of Meteorin can stimulate satellite glia to induce a morphological change in the PNS [71], its full function in the periphery remains to be determined. We report a necessary in vivo role of Meteorin in the differentiation of peripheral CNG through Jak/Stat3 signaling. Taken together, CNG display key features of astroglial populations.

In addition to identifying a cardiac glial population, we have also elucidated further neural crest contribution to the heart. During development of the zebrafish, 2 waves of neural crest cells populate the heart [60]. The first wave occurs between 24 and 30 hpf and contributes to the cardiomyocyte population. This is followed by a second wave at 80 hpf that recruits FGF signaling to populate the OT. The identity of the neural crest cells in the second wave is currently unknown, with Cavanaugh and colleagues [60] suggesting that the cells may contribute to the smooth muscle of the OT and Abdul-Wajid and colleagues [81] confirming that the cells are not cardiomyocytes. Although there have been recent reports of a GFAP-progenitor population that can give rise to vascular smooth muscle and endothelial cells in the heart [82], our data demonstrating that a subset of the *gfap*^*+*^ population also expresses the astrocyte-specific gene, *glast*/*slc1a3b*, and uses an astroglial determinant suggest a glial identity. Furthermore, while Cavanaugh and colleagues [60] and Abdul-Wajid and colleagues [81] report an estimate of around 15 to 20 neural crest cells throughout the heart by 4 dpf, our work shows that there are only an average of 2.5 *gfap*^*+*^ cells primarily localizing to the OT by that same time point. We therefore propose that a population of the neural crest cells contribute to the CNG population, and the remaining neural crest cells differentiate into cardiomyocytes and smooth muscle cells. The diversity in cellular fate would also explain why Cavanaugh and colleagues [60] observed a decrease in heart rate after neural crest ablation as compared to the increase in heart rate we find after *gfap*^*+*^ ablation, given that many cellular populations would be affected rather than just the CNG population.

Since the neural crest population of the OT remains unidentified, the discovery that neural crest–derived *gfap*^*+*^ cells in the OT modulate embryonic heart rate is especially interesting. In mammals, this neural crest population has been shown to impact remodeling of the OT as well as patterning of the great vessels exiting the heart [35–37]. The severity of neural crest deficiency is directly associated with the severity of OT complications [40], which is crucial since 30% of CHDs are due to OT defects [37]. While the structural role of neural crest cells in the OT has been well studied [83], the functional role of the neural crest cells in the OT and valves have not yet been fully elucidated. However, some research suggests that these cells contribute to the cardiac conduction system (CCS) by regulating the lamellar compaction of the bundle of His and insulating electrical propagating fibers [35]. Furthermore, a study found that a subpopulation of these cells in the CCS is labeled with the immature glial markers, BLBP and GLAST [36]. However, confirmation of a glial identity was never determined. Therefore, our finding of CNG could potentially fill the long-standing gap in the cellular identification of the neural crest cells that contribute to OT formation and CCS function.

This spatial control of heart function by glia is additionally impactful as it may contribute to the pathophysiology of idiopathic ventricular arrhythmias [84], which is poorly understood. In the absence of structural heart disease, it is thought that improper cAMP-mediated signaling of the right ventricular outflow tract (RVOT) comprises the majority of idiopathic ventricular arrhythmias [85]. It has been suggested that increased cAMP signaling elevates levels of intracellular Ca^2+^ to induce a delayed after depolarization (DAD) of CCS action potentials, specifically Purkinje fibers [86–88]. This results in ventricular tachycardia and can ultimately lead to ventricular fibrillation [74], which is lethal. In the enteric nervous system (ENS), enteric glia act as a primary contributor to cAMP production and signaling in the myenteric plexus [89], where the ENS regulates GI tract motility. Furthermore, increasing Ca^2+^ activity in enteric glia leads to increased contractions of the gut through excitatory neuronal signaling [27]. Given our knowledge of enteric glia, it is thus possible that CNGs impact heart function in a similar manner. However, more research is necessary to know how CNGs impact the ionic activity of the cardiac environment to regulate heart function.

In addition to Scherschel and colleagues’ finding that *s100b*^*+*^ cells impact recovery from catheter ablation [34], we have found that CNG are necessary for maintaining normal cardiac functions in the absence of disease. When taken into consideration with research on the role of enteric glia in gut motility and disease [26], these results raise the possibility that specialized glia exist across organs to impact function. Glial-like populations have been briefly described in the spleen [29], lungs [31,90], pancreas [30,91], and skin [32] and hint at important specialized functions per organ. Neural crest cells migrate along the neurovasculature to populate the spleen, where they express the astroglial genes *blbp*, *s100b*, and *gfap* [29]. While this population associates with immune cells and has been suggested to impact lymphocyte trafficking in the spleen [29], it has not been confirmed. Furthermore, the developing lung consists of neural crest–derived *gfap*^*+*^ cells that localize to bronchioles and pulmonary vasculature in a net-like pattern [31,90]. The pancreas and skin have also been described to have astroglia-like cells with a neural crest origin, although they are termed as specialized nonmyelinating Schwann cells. Peri-islet Schwann cells in the pancreas support individual endocrine regions of hormone-producing islets, and gliosis of these cells is implicated in diabetes pathology [30,91]. Recently, the skin has been found to have nociceptive Schwann cells, which drives pain in response to mechanical stimuli [32]. Our findings of CNG taken together with the above data indicate an extensive underexplored network of organ-associated glia that have functional roles dependent upon the environment. Further understanding of these specialized astroglial populations is therefore necessary, given their potential impact on organ physiology.

While our study reveals an undescribed glial population in the heart, there are current limitations to the present results. Here, we report that the *gfap*^*+*^ population serves to either inhibit or delay internal neuronal development. While axons are only present in the absence of glia, this contradicts previous literature placing neuronal development before astroglial development [19]. Furthermore, Nishino and colleagues found that Meteorin signaling promotes rather than inhibits axon extension [71]. A tertiary hypothesis to potentially explain this discrepancy is that ablation of the *gfap*^*+*^ cells leads to a reactive gliosis effect similar to what is seen in astrocytes [92], and a release of gliosis factors promotes overextension of extrinsic innervation of sympathetic or parasympathetic axons. However, due to the current lack of both visualization tools and knowledge of early ICNS development, more information is necessary to fully elucidate the role that glia play in cardiac neuronal development. In addition, our present research shows that CNG cells regulate heart function through ANS modulation, but cells in the ICNS also have the potential to impact surrounding cardiomyocytes. Therefore, further insight into the extent of CNG modulation of cardiomyocytes is necessary to understand the role of glia in ventricular arrhythmias. In addition, while our study identifies *metrn* expression in cardiac cells in mouse and humans and can be detected in the OT, the role of METRN/Metrn in heart physiology of mouse and humans is not known. Even though METRN/Metrn is detected in embryonic mouse and human cells, this study only investigates the location, morphology, origin, determinant, and physiological function of nexus glia in zebrafish.

## Conclusions

Taken together, this study provides developmental, molecular, and functional characterization of nexus glia that are integral to cardiac health. Collectively, the above data demonstrate that an astrocyte-like cell, termed nexus glia, populates cardiac tissue across species and impacts embryonic heart rate and rhythm. This introduces the possibility that other visceral organs may also have a nexus glia–like population that is crucial to function.

## Methods

### Experimental model and subject details

All experimental procedures adhered to the NIH guide for the care and use of laboratory animals and was approved by The University of Notre Dame Institutional Animal Care and Use Committee approved all animal studies (protocol 19-08-5464). Notre Dame IACUC adheres to the United States Department of Agriculture, the Animal Welfare Act (USA), and the Assessment and Accreditation of Laboratory Animal Care International.

#### Zebrafish

Zebrafish stable strains used in this study were AB, *Tg(gfap*:*nuc-gfp)* [93], *Tg(gfap*:*gfp)* [44], *Tg(gfap*:*nsfb-mcherry)* [59,94], *Tg(sox10*:*nls-Eos)* [62], *Tg(sox10*:*gal4 + cmcl2*:*gfp)* [95], *Tg(gfap*:*nuc-gfp);metrn*^*−/−*^, *Tg(slc1a3b*:*myrGCaMP6-P2A-H2AmCherry)* [43], and *Tg(slc1a3b*:*myrGFP-P2A-H2AmCherry)* [43]. Zebrafish embryos were produced through pairwise mating and placed in an incubator at 28°C in constant darkness [96]. At 2 dpf, embryos were treated with PTU (0.003%) to reduce pigmentation.

Adult zebrafish were anesthetized with 3-amino-benzoic acid ester (Tricaine) before heart dissection. The transgenic hearts used in this study were AB, *Tg(gfap*:*nuc-gfp)*, and *Tg(gfap*:*gfp)*. A ventral midline incision was made to expose the heart, and the bulbus arteriosus, ventricle, and atrium were removed. Hearts were immediately fixed in 4% paraformaldehyde (PFA).

#### Mouse

Mice were housed socially in a temperature-controlled room with a 12-hour light–dark cycle. They were fed a chow diet. All caretaking and experiments were approved by the University of Notre Dame Institutional Animal Care and Use Committee (IACUC). Mice at the endpoint were euthanized by carbon dioxide asphyxiation in a Euthanex system chamber. In total, 10 adult mouse hearts were dissected and immediately fixed in 4% PFA. Hearts were prepared for cryosection by washing 3 times with PBS for 30 minutes, then incubated in 5% sucrose solution for 30 minutes. Hearts were then placed in 10%, 20%, and 30% sucrose overnight in succession at 4°C. Next, hearts were placed in a mold and covered with OCT freezing medium and placed in −80°C until cryosectioning (32 μm sections). Both male and female hearts were used in all experiments. Mouse adult single-cell sequencing data were extracted from Skelley and colleagues [66].

#### Human

Slices of human tissue from 2 donated adult hearts were provided by Dr. Pinar Zorlutuna, who obtained them from the Indiana Donor Network. Deidentified human hearts that were deemed unsuitable for transplantation and donated to research were acquired from Indiana Donor Network under the Institutional Review Board (IRB) approval for deceased donor tissue recovery. Left ventricles from 40- and 63-year-old humans were fixed with PFA (4%) and cryosectioned (10 μm sections). Slices were immediately transferred to superfrost glass slides and placed in −80°C until used.

### Single-cell sequencing analysis

#### Mouse

A matrix of raw UMI counts for the non-myocyte cardiac cells profiled via single-cell RNA sequencing in Skelly and colleagues was obtained from the EMBL-EBI ArrayExpress database (E-MTAB-6173). Analysis was conducted in R using Seurat (v3) [97]. Cells with fewer than 200 or greater than 5,000 genes detected, cells with greater than 20,000 UMIs, and cells with greater than 25% of UMIs mapping to the mitochondrial genome were excluded from downstream analysis to remove potential doublets and unhealthy or poorly sequenced cells. Raw counts were log normalized (UMI counts per gene per cell were divided by the total number of reads, multiplied by a scale factor of 10,000, and natural log transformed; thus, ln(TPT + 1), where TPT is transcripts per 10,000). The top 2,000 variable features were calculated, and gene expression was scaled using the ScaleData function, regressing out the percentage of reads mapping to the mitochondrial genome per cell. PCA was performed using the top variable features, and k-means clustering (with a resolution parameter of 0.3) and dimensionality reduction via t-SNE were performed using 11 principal components. This yielded 12 clusters, 4 of which highly expressed typical fibroblast marker genes such as *Col1a1* and were thus labeled fibroblasts. We then subclustered these 4 clusters, calculating new top 2,000 variable features among the selected cells, and scaled, and performed PCA, dimensionality reduction via t-SNE, and k-means clustering as described above (using 12 principal components and a resolution of 0.6). This resulted in 6 clusters, one of which had elevated expression of *S100b*. In Fig 5C, this *S100b*+ cluster is denoted as “subcluster,” while the other 5 clusters are together denoted as “main cluster.” Plots overlaying log-normalized gene expression onto the t-SNE plot were created using Seurat. Differential expression testing between clusters was conducted using Seurat, with thresholds of significance of ±0.25 natural log-fold change and *p* < 0.05. The R code necessary for Skelly and colleagues analysis is in S3 Data.

#### Human

Embryonic sections were directly taken from the Asp and colleagues database (https://hdca-sweden.scilifelab.se/a-study-on-human-heart-development/), and section 3 was used for all genes [64]. Human embryonic single-cell sequencing data of *metrn* were similarly extracted from the Asp and colleagues database, and we report data from all 14 clusters. Both spatial and single-cell sequencing data from Asp and colleagues were examined at 6.5 weeks postconception (PCW) [64]. All figures panels that utilized Asp and colleagues were synthesized on the online portal for that paper and imported into the figures unedited.

Human adult single-cell sequencing data were directly extracted from the neuronal cluster of Litviňuková and colleagues [65]. All figure panels that represented data from Litviňuková and colleagues were generated from the online portal for that paper and imported in the figured unedited.

### Generation of *metrn* mutant

*Tg(gfap*:*nucgfp)* animals at the one-cell stage were injected with a CRISPR cocktail composed of a guide RNA and Cas9 protein. The gRNA duplex of trRNA/crRNA were separately synthesized by IDT. The gRNA sequence targeted 5′- GGATTTCATTCCTGACGGGT -3′ in Exon 1 and was identified with chopchop [98]. EnGen Cas9 NLS, *S*. *pyogenes* from New England Biolabs was used. Injected animals were screened for disruption of GFP^+^ cells in the heart and then grown to adulthood. To identify potential *metrn* mutants, F_0_ injected adults were crossed to *Tg(gfap*:*nucgfp)* animals, and the resulting F_1_ progeny were genotyped. Genotyping of the initial F_1_ animals were done in a pool, with 12 individual animals represented in the pool. A 272-bp genomic region surrounding the PAM site was amplified, and sequencing with Sanger sequencing was performed. Genotyping for *metrn*^*−/−*^ genomic DNA was amplified using the following primers: 5′- GAGAAGCAGTGACCGAGAC -3′ and 5′- TCTGCTGTCTTGCATGATTTCT -3′. Pooled DNA that displayed potential heterogeneity among the sequence was then pursued as potential mutants. Such F_1_ animals were then grown to adulthood and fin clipped for genotyping. Genotyping was then performed by amplifying genomic DNA and followed by a DrdI digest (New England Biolabs, Cat # R0530), which cuts only the wild-type product. The length of the mutant band is 268 bp, and the length of the cut wild-type bands are 165 and 106 bp. All experiments in the study represent animals beyond the F_2_ generation.

### slc1a3b:nls-tdTomato construct

Creation of *slc1a3b*:*nls-tdTomato* construct was generated using the Gateway LR Clonase II Plus System (Thermo Fisher) using zebrafish-compatible Tol2 vectors [99]. A *p5e-slc1a3b* vector (gift from Kelly Monk) was used to drive expression in astroglial cells. For expression of nuclear tdTomato, we used *pMe-nlsTdTomato* and *p3e-polyA*. Multiple site gateway recombination of these vectors was accomplished using linearized *pDestTol2*.

### In vivo imaging

Larvae zebrafish were anesthetized with Tricaine and covered with 0.8% low-melting point agar. Zebrafish were mounted ventrally, with tails lifted dorsally and hearts flat against the glass cover of 35 mm Petri dishes. Images were acquired on a custom built 3i spinning disc confocal microscope as previously described [100,101], with a Zeiss Axio Observer Z1 Advanced Mariana Microscope; X-cite 120LED White Light LED System; filter cubes for GFP and mRFP; a motorized X,Y stage; a piezo Z stage; 20× Air (0.50 NA), 63× (1.15 NA), 40× (1.1 NA) objectives; CSU-W1 T2 Spinning Disk Confocal Head (50 μM) with 1× camera adapter and an iXon3 1Kx1K EMCCD camera; dichroic mirrors for 446, 515, 561, 405, 488, 561, and 640 excitation; laser stack with 405 nm, 445 nm, 488 nm, 561 nm, and 637 nm with laser stack FiberSwitcher; photomanipulation from vector high-speed point scanner ablations at diffraction limited capacity; and Ablate Photoablation System (532 nm pulsed laser; pulse energy 60 J at 200 Hz). For live animals, laser power 10 was used to visualize transgenes. For fixed tissue, laser power 50 was used to visualize transgenes and antibodies. Confocal images were acquired using a 60-μm stack with a 1-μm step size and compiled maximum z-projection. Time-lapsed images were collected over 24 hours at 5-minute intervals using a 60-μm stack. Adobe Illustrator (https://www.adobe.com/products/illustrator.html; San Jose, CA), ImageJ (https://imagej.nih.gov/ij/download.html; Bethesda, MD), and Imaris (https://imaris.oxinst.com/; Concord, MA) were used to process images. The only enhancement to presented images were universal changes to brightness and contrast.

### Photoconversion of Eos to fate-map cells

Migration trajectory of neural crest cardiac cells: *Tg(sox10*:*nls-eos)* 24 hpf embryos were used to perform all photoconversion experiments. Individual cell trajectories were mapped using 24-hour time-lapse imaging with 5-minute intervals of the anterior neural crest and heart. The Imaris Tracking feature was used to select neural crest cells in the beginning of the time-lapse video. These cells were determined using the “spots” feature to set an intensity and diameter threshold. The cell migration path was then automatically tracked over time.

Contribution of neural crest cells to cardiac populations: *Eos*^*+*^ nuclei located cranially were individually and focally exposed to 405 nm UV light through the 3i vector system, by either drawing a selection circle around one cell or multiple at 24 hpf to photoconvert the *eos* protein from green to red. Cell migration was mapped using 24-hour time-lapse imaging with 5-minute intervals of cranially photoconverted cells and the heart. ImageJ was used to quantify the number of photoconverted cells in the heart.

Fate mapping cardiac nexus glia: Secondary experiments with *Tg(sox10*:*nls-eos); Tg(gfap*:*nuc-GFP)* 24 hpf embryos included initial photoconversion as well as re-photoconversion with 405 nm UV light through the 3i vector system of nuclei in the heart at 4 dpf [102,103]. Larvae were fixed at 6 dpf and stained with anti-GFP (1:200, chicken, Abcam) to discern between *eos*^*+*^ and *GFP*^*+*^ nuclei. ImageJ was used to quantify the number of *eos*^*+*^ and *GFP*^*+*^ nuclei in the heart.

Ablation of neural crest cells: Tertiary experiments with *Tg(sox10*:*nls-eos); Tg(gfap*:*nuc-GFP)* embryos included initial photoconversion with 405 nm UV light through the 3i vector system at 24 hpf of either hindbrain or trunk neural crest. Starting at 24 hpf to 36 hpf, *eos*^*+*^ cells were focally ablated as they were migrating using a confocal 3i Ablate laser as previously described [100]. Larvae were fixed at 6 dpf and stained with anti-GFP (1:200, chicken, Abcam) to discern between *eos*^*+*^ and *GFP*^*+*^ nuclei. ImageJ was used to quantify the number of *GFP*^*+*^ nuclei in the heart.

### MTZ ablation of *gfap*^*+*^ cells

NTR-tagged zebrafish under a *gfap* promoter, *Tg(gfap*:*nfsb-mcherry)*, were used to perform *gfap*-specific drug ablations [94]. MTZ (Fisher Scientific, Cat# AC210340050) was diluted to 10 mM and contained in a dark container to avoid exposure to light. MTZ was injected into the caudal vein, leading directly to the heart, at 3 to 5 dpf. To confirm cardiac-specific ablation, mcherry intensity levels were measured in CNS *gfap*^*+*^ cells between MTZ and DMSO larvae. Only larvae with no change in mcherry intensity of CNS *gfap*^*+*^ cells between groups were used. To further confirm ablation of *gfap*^*+*^, mcherry intensity was measured in the hearts of MTZ-treated larvae. Quantifications were performed on larvae with decreased mcherry intensity.

### Laser ablation

*Tg(gfap*:*nuc-GFP)* 5 dpf embryos were anesthetized with Tricaine and covered with 0.8% low-melting point agar. Zebrafish were mounted ventrally, with tails lifted dorsally and hearts flat against the glass cover of 35 mm Petri dishes. All focal cell ablations were performed with previously described parameters [100]. In location-specific ablation experiments, ablations were performed solely at the bulbous arteriosus, ventricle, or atrium. In full ablation experiments, ablations were performed at all 3 sites. Control ablations were performed on non-*gfap*^*+*^ locations in the cardiac chamber of interest.

### Drug treatments

All embryos were dechorionated at 24 hpf and incubated in 3 mL egg water treated with 0.003% PTU until desired treatment time. All controls were performed with 1% DMSO in egg water.

Pharmacological treatments: The chemical reagents used in this experiment were S31-201 (400 μM, Sigma-Aldrich, Cat# 573130) [104], CAS 457081-03-7 (1 μM, Sigma-Aldrich, Cat# CC1000), and SC144 (1 μM, Sigma-Aldrich, Cat# 5.06387.0001) [105]. For the S31-201 wash-in/wash-out experiment, larvae were treated with 400 μM S31-201 at 3 dpf and washed out at either 4 dpf or 5 dpf. Larvae were also treated with S31-201 either at 3 dpf, 4 dpf, or 5 dpf. Both groups were imaged at 6 dpf. Larvae at 4 dpf were treated with either 400 μM S31-201, 1 μM CAS 457081-03-7, or 1 μM Sc144 and imaged at 6 dpf.

Sympathetic and parasympathetic manipulation: The chemical reagents used in this experiment were isoprenaline hydrochloride (100 μM, isoproterenol, Sigma-Aldrich, Cat# I5627) [57] and carbamoylchloride (500 μM, carbachol, Sigma-Aldrich, Cat# C4382) [57]. Treatment with either 100 μM isoproterenol or 500 μM carbachol was performed immediately after *gfap*^*+*^ ablations at 6 dpf and imaged 1 hour post-ablation.

### Immunohistochemistry

The same immunohistochemistry protocol was performed on all zebrafish, mouse, and heart tissue samples. The primary antibodies used in initial cardiac glial screening were anti-GFAP (1:250, rabbit, DAKO), anti-Vimentin (1:500, mouse, DSHB), anti-NG2 (1:250, mouse, Millipore), anti-MBP (1:50, rabbit, Appel lab), anti-MEF2 (1:200, mouse, Santa Cruz), and anti-MF20 (1:20, chicken, DSHB). The primary antibodies used in neuronal and synaptic identification were anti-AcTub (1:200, mouse, Sigma-Aldrich), anti-HuCD (1:200, mouse, Thermo Fisher), anti-Znp-1 (1:500, mouse, DSHB), and anti-SV2 (1:100, mouse, DSHB). The antibody used in photoconversion experiments was anti-GFP (1:200, chicken, Abcam). The secondary antibodies used were Alexa Fluor 594 goat anti-mouse (1:600, Thermo Fisher), Alexa Fluor 647 goat anti-rabbit (1:600, Thermo Fisher), and Alexa Fluor 647 goat anti-chicken (1:600, Thermo Fisher). Modified immunohistochemistry protocols were adapted from Inoue and Wittbrodt (2011) and the Chen Lab at Stanford [106]. Larvae were fixed using 4% PFA in PBST (PBS, 0.25% TritonX-100) at 4°C overnight. Larvae were then washed with 25%, 50%, and 100% methanol at 10 minutes each, and left in 100% methanol at −20°C overnight. Larvae were then rehydrated with 50% and 25% methanol at 10 minutes each, then washed with PBST 3 times for 10 minutes. Larvae were next washed with 150 mM Tris-HCl (pH = 9.0) for 5 minutes at room temperature and then heated at 70°C for 15 minutes. Larvae were then incubated with cold acetone and chilled at −20°C for 20 minutes and again washed with PBST 3 times for 10 minutes. Next, larvae were incubated for an hour in 5% goat serum in PBST at 25°C. Larvae were then incubated in 5% goat serum in PBST with the primary antibody for an hour at 25°C and then incubated overnight at 4°C. After 3 washes of PBST for 30 minutes each, the larvae were then incubated in 5% goat serum in PBST with the secondary antibody at 25°C for an hour and then transferred to 4°C overnight. The larvae were then washed 3 times with PBST for 30 minutes and finally transferred to a 50% glycerol stock and stored in 4°C until imaging.

### Whole-mount larval zebrafish RNAScope

The following ACD RNAscope probes were used: *metrn* (1:50, 80 μL, C3, ACD), *id1* (1:50, 80 μL, C1, ACD), and *cdh11* (1:50, 80 μL, C1, ACD). Larvae were fixed in 4% PFA at 25°C for 30 minutes, then transferred to a new eppendorf tube and dehydrated with 25%, 50%, and 100% methanol at 10 minutes each. Larvae were left in 100% methanol at −20°C overnight and then rehydrated with 50% and 25% methanol in PBSTw (PBS, 0.1% Tween-20) at 10 minutes each. Next, larvae were air dried for 30 minutes, then washed twice in PBSTw for 5 minutes. Larvae were then permeabilized in proteinase K (10 mg/mL, 1:2,000 in PBSTw) at 25°C for 6 minutes and immediately washed 3 times with PBSTw for 10 minutes at 25°C. Larvae were then incubated with the probe (2 drops of C1 or 80 μL of C2/C3 in diluent buffer) at 40°C overnight. After incubation, larvae were washed twice with SSCTw (saline-sodium citrate buffer, 0.1% Tween-20) and then washed with 4% PFA for 10 minutes at 25°C. Larvae were transferred to a new eppendorf tube and washed 3 times with SSCTw for 10 minutes each at 25°C. Next, larvae went through a series of incubations in 2 drops of Amp1 at 40°C for 30 minutes, 2 drops of Amp2 at 40°C for 30 minutes, 2 drops of Amp3 at 40°C for 15 minutes, 2 drops of HRP-C1/2/3 at 40°C for 30 minutes, opal fluorophore 650 (1:500) at 40°C for 30 minutes, and 2 drops of Multiplex FLv2 HRP blocker at 40°C for 30 minutes. Larvae were washed twice with SSCTw at 25°C for 10 minutes between each incubation. Fish were then incubated in 2 drops of DAPI at 4°C, washed twice with SSCTw at 25°C for 10 minutes, and finally stored in 50% glycerol and stored in 4°C until imaging [107].

### Colocalization quantifications

Axonal and GFAP branch localization: Initial colocalization was quantified by separating axons into 3 different branches. The main branch stemming from the cell body was characterized as the primary branch, the axonal branches stemming off of the primary branch were considered secondary, and the axonal branches off of the secondary branch were deemed tertiary. Localization was determined by overlap in intensity of AcTub and GFAP (see below). ImageJ was used to count the number of *gfap*^*+*^ processes that localized with each branch category and was quantified as the average percentage of *gfap*^*+*^ localization per branch.

Intensity localization: The quantification of colocalization utilized the intensity function on ImageJ. A 3D rendering of *actub*^*+*^ axons and *gfap*^*+*^ projections was made using 3i Slidebook software (https://www.intelligent-imaging.com/slidebook; Denver, CO) and rotated 90°. In ImageJ, a line was drawn through the *actub*^*+*^ axons (blue channel) and *gfap*^*+*^ projections (green channel), and intensity over distance of both channels was measured to determine the area of colocalization.

### Heart morphology quantification

Wild-type and *metrn*^*−/−*^ animals were mounted in cooled 0.8% low-melting point agar at 6 dpf, with tails lifted dorsally and hearts flat against the glass cover of 35 mm Petri dishes. Embryonic heart morphology was then captured in 60 μm stacks using bright-field on a spinning disc confocal. The freehand tool on ImageJ was used to separately trace the chambers of the heart at the same physiological time point, when the ventricle was at its highest point of relaxation. The shape descriptor plug-in on ImageJ was used to calculate circularity, roundness, solidity, and aspect ratio of each chamber.

### Heart function quantification

After manipulations were performed as described above, zebrafish were mounted in cooled 0.8% low-melting point agar at 6 dpf, with tails lifted dorsally and hearts flat against the glass cover of 35 mm Petri dishes. No anesthetic was used in order to precisely capture heart rate. Embryonic heart function was then examined using bright-field on a spinning disc confocal and was recorded at 30 frames per second over a 1-minute period. Movies were then converted into an image sequence for analysis in ImageJ. A straight line with a 5-μm width was drawn through the diameter of the ventricle and the multi-kymograph function was used to plot intensity changes over time. Change in intensity of the ventricle indicates when blood flow has entered (lower values) and left (higher values) the ventricle. Heart rate was then quantified by scoring the number of full ventricular contractions in a 1-minute period. Heart rhythm was also measured over a 1-minute period. The average of the maximum and minimum values was calculated to create a median threshold line. Data peaks on or below the threshold line that lasted 5 frames or longer were considered ventricular fibrillation. The total frames were then added and divided by 30 to calculate duration in seconds.

### Blind quantifications and statistical analysis

Scientists were blind to the identity of the experimental versus control group during quantifications. Experiments with drug treatments were performed by the primary scientist and given to a secondary scientist, and heart function was confirmed in a blinded fashion. Experiments with *metrn* mutants were quantified before genotyping, and matching of phenotype and genotype was done during figure generation. Normality distribution of experiments was assessed using the “Normality and Lognormality Test” feature on Prism, and Gaussian distribution was assessed followed by a D’Agostino–Pearson omnibus test. When applicable, a one-way ANOVA was performed, followed by Tukey’s post hoc test to account for variance.

### Software

Imaris, Adobe Illustrator, Slidebook, Prism, and ImageJ were used in this study to analyze, acquire, or compile figures.

## Supporting information

S1 Fig *gfap*^*+*^ cells do not express pericyte or fibroblast markers. (A) Quantification of the number of *gfap*:*nucGFP*^***+***^ cells that express *slc1a3b*:*nls-tdTomato* (*n =* 124 cells) or GS (*n* = 76 cells). (B) Quantification of number of *gfap*:*nucGFP*^***+***^/Mef-2A^***+***^ cells per 6 dpf (*n* = 211 cells) and adult ventricles (*n* = 49 cells). (C) Confocal maximum z-projection of adult whole-mount zebrafish heart stained with NG2 and GFAP. The *gfap*^*+*^ cells (white arrowhead) do not express NG2 (white arrow). (D) Confocal maximum z-projection of adult sectioned mouse hearts stained with Vimentin and GFAP. One population expresses both Vimentin and GFAP (white arrowhead), and another population expresses only GFAP (white arrows). Scale bar equals 10 μm. GS, glutamine synthetase.
(TIF)

S2 Fig *metrn*^*−/−*^ hearts do not have morphological changes. (A) Schematic representation of wild-type blood flow intensity over time of the ventricle. High intensity indicates contraction of the ventricle, and low intensity indicates relaxation of the ventricle. (B) Shape descriptor quantifications of the ventricular in wild-type and *metrn*^*−/−*^ animals. (C) Shape descriptor quantifications of the outflow tract in wild-type and *metrn*^*−/−*^ animals. (D) Shape descriptor quantifications of the atrium shape in wild-type and *metrn*^*−/−*^ animals.
(TIF)

S1 Table List of key resources and reagents. Shown are a list of reagents, sources, and identifier information for all reagents used in this study.
(PDF)

S2 Table Table of statistical information for each figure. A summary of statistical information for each figure pane in the manuscript.
(PDF)

S1 Movie Video of a beating wild-type heart. Video of the heart of wild-type animals. Images were captured at 30 frames/second.
(MP4)

S2 Movie Video of a beating *metrn*^*+/−*^ heart. Video of the heart of *metrn*^***+/−***^ animals. Images were captured at 30 frames/second.
(MP4)

S3 Movie Video of a beating metrn^*−/−*^ heart. Video of the heart of *metrn*^***−/−***^ animals. Images were captured at 30 frames/second.
(MP4)

S4 Movie Video of a beating wild-type heart with ablation of *gfap*^*+*^ cells in the outflow tract. Video of the heart of animals in which *gfap*^*+*^ cells were spatially ablated in the outflow tract. Images were captured at 30 frames/second.
(MP4)

S5 Movie Video of a beating wild-type heart with ablation of *gfap*^*+*^ cells in the ventricle. Video of the heart of animals in which *gfap*^*+*^ cells were spatially ablated in the ventricle. Images were captured at 30 frames/second.
(MP4)

S6 Movie Video of a beating wild-type heart with ablation of *gfap*^*+*^ cells in the atrium. Video of the heart of animals in which *gfap*^*+*^ cells were spatially ablated in the atrium. Images were captured at 30 frames/second.
(MP4)

S1 Data Raw data of the data represented in this manuscript. All raw data and statistical values represented in different spreadsheet tabs per figure panel.
(XLSX)

S2 Data Analysis of scRNA sequencing from Skelly and colleagues that is referenced in the figure. Spreadsheet tab shows enriched genes that are expressed in the fibroblast subcluster from Skelly and colleagues.
(XLSX)

S3 Data R code for analysis of Skelly and colleagues. Detailed R code for the analysis of the Skelly and colleagues dataset that is reported in this manuscript.
(TXT)

## Abbreviations

AcTub: acetylated tubulin
ANS: autonomic nervous system; bpm, beats per minute
CCS: cardiac conduction system
CHD: congenital heart disease
CNG: cardiac nexus glia
CNS: central nervous system
DAD: delayed after depolarization
dpf: days postfertilization
ENS: enteric nervous system
hpf: hours postfertilization
ICNS: intracardiac nervous system
MBP: myelin basic protein
mpf: months postfertilization
MTZ: metronidazole
NTR: nitroreductase
OT: outflow tract
p-Eos: photoconverted-Eos
PNS: peripheral nervous system
PSNS: parasympathetic nervous system
RVOT: right ventricular outflow tract
SNS: sympathetic nervous system
