# Supplementary figures and images for: Identification of astroglia-like cardiac nexus glia that are critical regulators of cardiac development and function
Source: PLoS Biol. 2021 Nov 18;19(11):e3001444. doi: 10.1371/journal.pbio.3001444 (PMC8601506; doi:10.1371/journal.pbio.3001444)

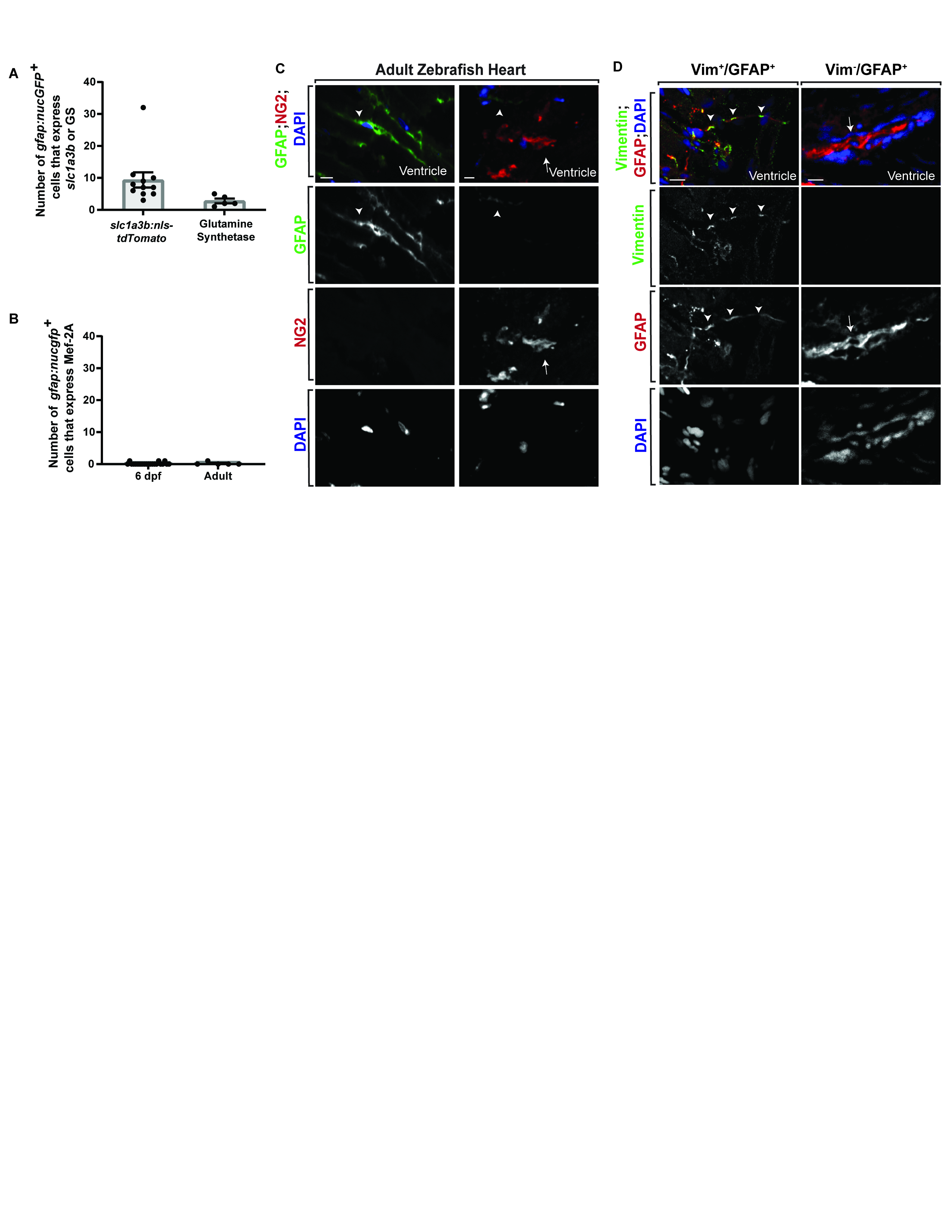

Supplement: S1 Fig — (A) Quantification of the number of gfap:nucGFP+ cells that express slc1a3b:nls-tdTomato (n = 124 cells) or GS (n = 76 cells). (B) Quantification of number of gfap:nucGFP+/Mef-2A+ cells per 6 dpf (n = 211 cells) and adult ventricles (n = 49 cells). (C) Confocal maximum z-projection of adult whole-mount zebrafish heart stained with NG2 and GFAP. The gfap+ cells (white arrowhead) do not express NG2 (white arrow). (D) Confocal maximum z-projection of adult sectioned mouse hearts stained with Vimentin and GFAP. One population expresses both Vimentin and GFAP (white arrowhead), and another population expresses only GFAP (white arrows). Scale bar equals 10 μm. GS, glutamine synthetase. (TIF) [file pbio.3001444.s001.tif]

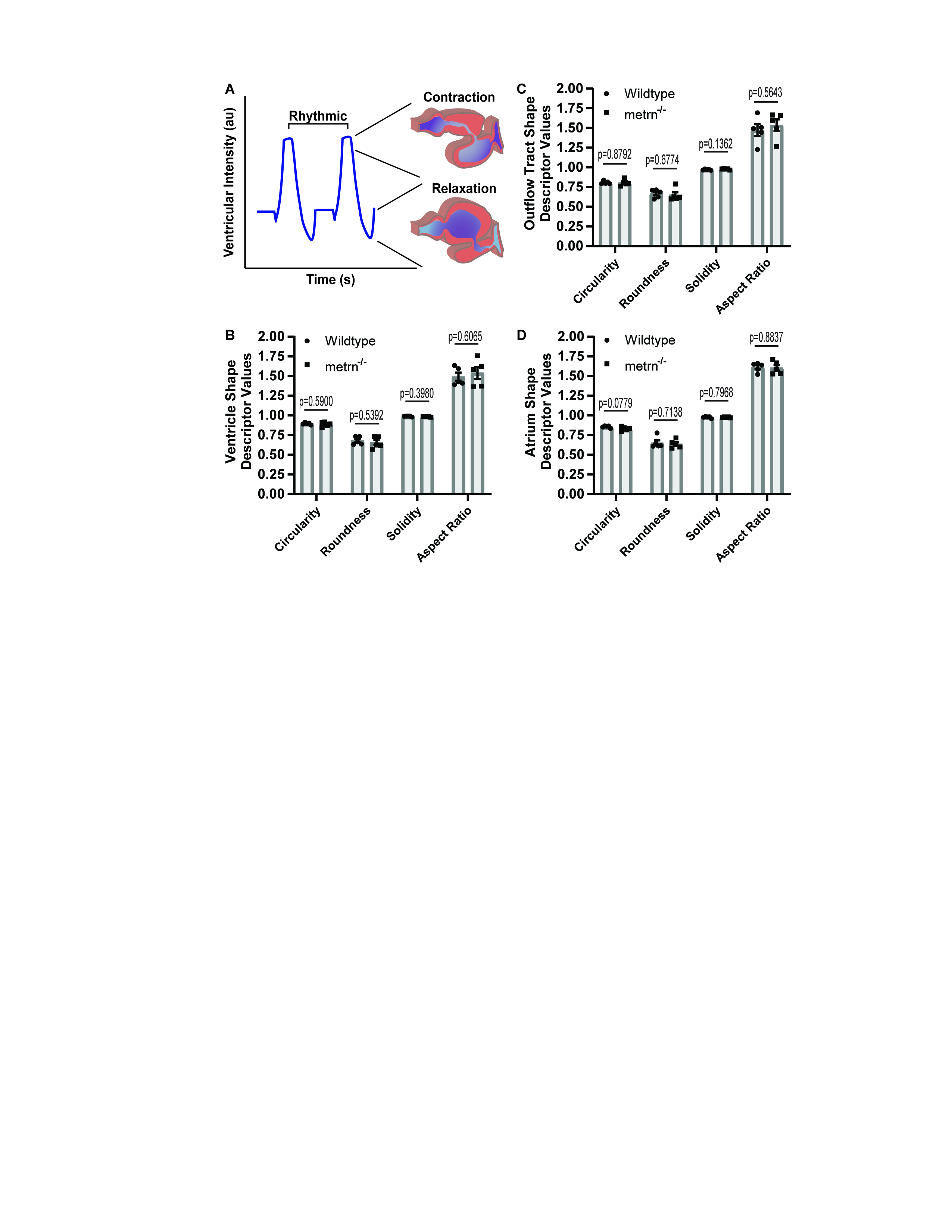

Supplement: S2 Fig — (A) Schematic representation of wild-type blood flow intensity over time of the ventricle. High intensity indicates contraction of the ventricle, and low intensity indicates relaxation of the ventricle. (B) Shape descriptor quantifications of the ventricular in wild-type and metrn−/− animals. (C) Shape descriptor quantifications of the outflow tract in wild-type and metrn−/− animals. (D) Shape descriptor quantifications of the atrium shape in wild-type and metrn−/− animals. (TIF) [file pbio.3001444.s002.tif]
