# Supplementary material for: Identification of astroglia-like cardiac nexus glia that are critical regulators of cardiac development and function
Source: PLoS Biol. 2021 Nov 18;19(11):e3001444. doi: 10.1371/journal.pbio.3001444 (PMC8601506; doi:10.1371/journal.pbio.3001444)
Supplement: S1 Table — Shown are a list of reagents, sources, and identifier information for all reagents used in this study. (PDF) [file pbio.3001444.s003.pdf]

Table S1. List of key resources and reagents.

REAGENT or RESOURCE	SOURCE	IDENTIFIER
Antibodies		
Rabbit anti-GFAP	DAKO	Cat #: Z0334
Rabbit anti-MBP	Appel Lab	N/A
Mouse anti-NG2	Millipore	Cat #: AB5320
Mouse anti-Vimentin	DHSB	Cat #: H5
Mouse anti-MEF-2	Santa Cruz	Cat #: sc-17785
Chicken anti-MF20	DHSB	RRID: AB_2147781
Mouse anti-Acetylated Tubulin	Sigma-Aldrich	Cat #: T7451
Mouse anti-HuCD	Thermo Fisher	Cat #: A-21271
Mouse anti-SV2	DHSB	RRID: AB_2315387
Mouse anti-Znp-1 (synaptotagmin)	DHSB	RRID: AB_10013783
Chicken anti-GFP	Abcam	Cat #: ab13970
Alexa Fluor 560 goat anti-mouse	Thermo Fisher	Cat #: A-11032; RRID: AB_2535767
Alexa Fluor 647 goat anti-rabbit	Thermo Fisher	Cat #: A-20991; RRID: AB_2535814
Alexa Fluor 647 goat anti-chicken	Thermo Fisher	Cat #: A-21449; RRID: AB_2535866
RNAScope		
RNAscope Multiplex Fluorescent Reagent Kit v2	Advanced Cell Diagnostics	Cat #: 323100
RNAScope Probe-*metrn*	Advanced Cell Diagnostics	This paper
RNAScope Probe-*id1*	Advanced Cell Diagnostics	Cat # :517531-C1
RNAScope Probe-*cdh11*	Advanced Cell Diagnostics	Cat #: 530551-C1
Opal 650 Fluorophore	Akoya Biosciences	Cat #: FP1496001KT
Chemicals		
S31-201	Sigma-Aldrich	Cat #: 573130
CAS 457081-03-7	Sigma-Aldrich	Cat #: CC1000
Sc144	Sigma-Aldrich	Cat #: 5.06387.0001
Isoproterenol	Sigma-Aldrich	Cat #: I5627
Carbamoylchloridem (Carbachol)	Sigma-Aldrich	Cat #: C4382

Experimental Models: Organisms/Strains		
AB	N/A	N/A
Zebrafish: *Tg(sox10:nls-Eos)*	Curran et al. 2010	ZFIN: ZDB-ALT-110721-2
Zebrafish: *Tg(gfap:nucGFP)*	Bernadardos et al. 2007	ZFIN: ZDB-TGCONSTRCT-070830-1
Zebrafish: *Tg(gfap:gfp)*	Bernardos et al. 2006	ZFIN: ZBD-ALT-060623-4
Zebrafish: *Tg(sox10:gal4-cmcl2:gfp)*	Hines et al. 2015	ZFIN: ZDB-FISH-150901-5454
Zebrafish: *Tg(gfap:nsfb-mcherry)*	Johnson et al. 2016	ZFIN: ZDB-ALT-160630-2
Zebrafish: *Tg(slc1a3b:myrGCaMP6-P2A-H2AmCherry)*	Chen et al. 2020	ZFIN: ZDB-TGCONSTRCT-200915-3
Zebrafish: *Tg(slc1a3b:myrGFP-P2A-H2AmCherry)*	Chen et al. 2020	ZFIN: ZDB-TGCONSTRCT-200915-2
Zebrafish: *metrn*$^{-/-}$	This paper	N/A
Mouse: Wildtype	Vaughn Lab, University of Notre Dame	N/A
Human: 40-year old heart, 63-year old heart	Zorlutuna Lab, University of Notre Dame	N/A
Mutation and Genotyping Reagents		
metrn gRNA sequence: GGATTTCATTCCTGACGGGT	ChopChop	This paper
Forward: 5’- GGAGAAGCAGTGACCGAGAC -3’	ChopChop	This paper
Reverse: 5’- TCTGCTGTCTTGCATGATTTCT -3’	ChopChop	This paper
DrdI	New England Biolabs	Cat #: R0530
Constructs		
slc1a3b:nls-tdTomato	This paper	N/A
Software and Algorithms		
ImageJ	NIH	RRID: SCR_003070; https://imagej.nih.gov/ij/
MTrackJ	Image Science	https://imagescience.org/meijering/software/mtrackj/
Imaris	Oxford Instruments	RRID: SCR_007370
Slidebook	3i	RRID: SCR_014300; https://www.intelligent-imaging.com/slidebook
Prism	GraphPad	RRID: SCR_002798; https://www.graphpad.com/
