# Supplementary material for: Identification of astroglia-like cardiac nexus glia that are critical regulators of cardiac development and function
Source: PLoS Biol. 2021 Nov 18;19(11):e3001444. doi: 10.1371/journal.pbio.3001444 (PMC8601506; doi:10.1371/journal.pbio.3001444)
Supplement: S2 Table — A summary of statistical information for each figure pane in the manuscript. (PDF) [file pbio.3001444.s004.pdf]

| Figure number | Number Reported (When applicable, number reported as Average +/- SEM) | n value<br>Number of Hearts | Pooled Cellular Number | | p-values | Statistical test |
|---|---|---|---|---|---|---|
| 1A | (Zebrafish) N/A | 10 hearts | N/A | N/A | N/A | N/A |
| | (Mouse) N/A | 6 hearts | N/A | N/A | N/A | N/A |
| | (Human) N/A | 2 hearts | N/A | N/A | N/A | N/A |
| 1B | N/A | 8 hearts | N/A | N/A | N/A | N/A |
| 1C | Type 1: 71.15 +/- 2.34 microns | 8 hearts (10 processes) | 13 | 5 | Type 1 vs 2 vs 3: p<0.0001 | One-Way ANOVA |
| | Type 2: 24.75 +/- 0.72 microns | 8 hearts (10 processes) | | 5 | Type 1 vs 2: p<0.0001 | One-way ANOVA followed by Tukey's post hoc test |
| | Type 3: 26.86 +/- 0.79 microns | 8 hearts (5 processes) | | 3 | Type 1 vs 3: p<0.0001 | One-way ANOVA followed by Tukey's post hoc test |
| | | | | | Type 2 vs 3: p=0.7240 | One-way ANOVA followed by Tukey's post hoc test |
| 1D | Type 1: 8.20 +/- 1.07 processes | 8 hearts | 13 | 5 | Type 1 vs 2 vs 3: p<0.0001 | One-Way ANOVA |
| | Type 2: 2.00 +/- 0.00 processes | 8 hearts | | 5 | Type 1 vs 2: p<0.0001 | One-way ANOVA followed by Tukey's post hoc test |
| | Type 3: 4.60 +/- 0.24 processes | 8 hearts | | 3 | Type 1 vs. 3: p=0.0044 | One-way ANOVA followed by Tukey's post hoc test |
| 1E | N/A | 3 hearts | N/A | N/A | N/A | N/A |
| 2A | N/A | 4 hearts | N/A | N/A | N/A | N/A |
| 2B | N/A | 5 hearts | N/A | N/A | N/A | N/A |
| 2C | N/A | 10 hearts | N/A | N/A | N/A | N/A |
| 2D | slc1a3b:nls-tdTomato: 81.12 +/- 5.05% | 11 hearts | 317 | 227 | N/A | N/A |
| | GS: 18.91 +/- 5.59% | 5 hearts | | 90 | N/A | N/A |
| 2E | slc1a3b:nls-tdTomato: 86.15 +/- 4.11% | 11 hearts | 247 | 218 | N/A | N/A |
| | GS: 93.33 +/- 6.67% | 5 hearts | | 29 | N/A | N/A |
| 2F | N/A | 6 hearts | N/A | N/A | N/A | N/A |
| 2G | N/A | 21 hearts | N/A | N/A | N/A | N/A |
| 2H | N/A | 5 hearts | N/A | N/A | N/A | N/A |
| 2I | 6 dpf: 0.90 +/- 0.52% gfap+/mef2a+ cells | 21 hearts | 260 | 211 | N/A | N/A |
| | Adult: 1.80 +/- 1.80% gfap+/mef2a+ cells | 5 hearts | | 49 | | |
| 2J | N/A | 7 hearts | N/A | N/A | N/A | N/A |
| 3A | N/A | 5 hearts | N/A | N/A | N/A | N/A |
| 3B | Primary: type 1=95.00 +/- 5.00% | 5 hearts | 5 | 5 | primary vs secondary: p=0.0004 | One-way ANOVA followed by Tukey's post hoc test |
| | Secondary: type 1=48.51 +/- 6.39% | | | | secondary vs tertiary: p=0.0035 | One-way ANOVA followed by Tukey's post hoc test |
| | Tertiary: type 1=18.29 +/- 3.68% | | | | All groups: p<0.0001 | One-Way ANOVA |
| 3C | N/A | 5 hearts | N/A | N/A | N/A | N/A |
| 3D | Representative AcTub Intensity Peak Distance: 2-6 microns | 1 heart | 1 | 1 | N/A | N/A |
| | Representative GFAP Intensity Peak Distance: 5-8 microns | | | | N/A | N/A |
| 3E | N/A | 5 hearts (SV2) 6 hearts (synaptotagmin) | 55 | 5 per heart | N/A | N/A |
| 3F | Heart DMSO: 1.48 +/- 0.07 au | 8 hearts | N/A | N/A | Heart DMSO vs MTZ: p<0.0001 | Unpaired t-test |
| | Heart MTZ Treated: 0.08 +/- 0.02 au | 8 hearts | N/A | N/A | | |
| | Retina DMSO: 1.51 +/- 0.09 au | 5 hearts | N/A | N/A | Retina DMSO vs MTZ: p=0.1499 | Unpaired t-test |
| | Retina MTZ Treated: 1.29 +/- 0.10 au | 5 hearts | N/A | N/A | | |
| 3G | DMSO: 0.25 +/- 0.16 axons | 8 hearts | N/A | N/A | DMSO vs. MTZ: p<0.0001 | Unpaired t-test |
| | MTZ: 6.88 +/- 0.30 axons | 8 hearts | | | | |
| | Control Ablation: 0.00 +/- 0.00 axons | 5 hearts | | | Control vs Laser: p<0.0001 | Unpaired t-test |
| | Laser Ablation: 7.25 +/- 1.03 axons | 4 hearts | | | | |
| 3H | N/A | DMSO: 8 hearts, MTZ: 8 hearts | N/A | N/A | N/A | N/A |
| | N/A | Control: 5 hearts, Laser: 4 hearts | N/A | N/A | N/A | N/A |
| 4A | N/A | N/A | N/A | N/A | N/A | N/A |
| 4B | N/A | 6 hearts per age group | N/A | N/A | N/A | N/A |
| 4C | 4 dpf: 2.08 +/- 0.40 nuclei | 13 hearts | 505 | 15 | 4 vs 5: p=0.1092 | One-way ANOVA followed by Tukey's post hoc test |
| | 5 dpf: 6.92 +/- 0.54 nuclei | 12 hearts | | 42 | 5 vs 6: p=0.0108 | One-way ANOVA followed by Tukey's post hoc test |
| | 6 dpf: 13.77 +/- 1.20 nuclei | 13 hearts | | 71 | 6 vs 2 mpf: p<0.0001 | One-way ANOVA followed by Tukey's post hoc test |
| | 2 mpf: 62.83 +/- 5.13 nuclei | 6 hearts | | 377 | All groups: p<0.0001 | One-Way ANOVA |
| 4D | 4 dpf: OT = 96.34%, V =3.66%, A=0% | 10 hearts | 374 | 49 | N/A | N/A |
| | 5 dpf: OT = 65.78%, V =34.22%, A=0% | 9 hearts | | 90 | | |
| | 6 dpf: OT = 35.25%, V =39.33%, A=25.42% | 10 hearts | | 235 | | |
| 4E | 4 dpf: 1.72 +/- 0.47 nuclei | 11 hearts | 210 | 19 | 4 vs 5: p=0.4729 | One-way ANOVA followed by Tukey's post hoc test |
| | 5 dpf: 3.31 +/- 0.81 nuclei | 13 hearts | | 43 | 5 vs 6: p=0.0031 | One-way ANOVA followed by Tukey's post hoc test |
| | 6 dpf: 7.40 +/- 0.91 nuclei | 20 hearts | | 148 | All groups: p<0.0001 | One-way ANOVA |
| 4F | 4 dpf: OT = 66.67%, V =33.33%, A=0.00% | 6 hearts | 46 | 8 | N/A | N/A |
| | 5 dpf: OT = 50.00%, V =37.50%, A=12.50% | 4 hearts | | 9 | | |
| | 6 dpf: OT = 41.67%, V =44.07%, A=14.26% | 9 hearts | | 29 | | |
| 4G | N/A | 6 hearts | ~30 | ~5 per heart | N/A | N/A |
| 4H | N/A | N/A | N/A | N/A | N/A | N/A |
| 4I | N/A | 6 hearts | N/A | N/A | N/A | N/A |
| 4J | Hindbrain derived = 81.87 +/- 1.27% | 6 hearts | 225 | ~37 per heart | hindbrain vs other: p<0.0001 | Unpaired t-test |
| | Other = 18.13 +/- 1.26% | | | | | |
| 4K | OT: 100% | 5 hearts | 29 | 9 | N/A (all same values) | N/A |
| | Ventricle: 100% | | | 14 | N/A (all same values) | N/A |
| | Atrioventricular Valve: 100% | | | 6 | N/A (all same values) | N/A |
| 4L | N/A | 5 hearts | N/A | N/A | N/A | N/A |
| 4M | Hindbrain: 1.67 +/- 0.88 | 3 hearts | 46 | 5 | Hindbrain vs Trunk: p=0.0023 | Unpaired t-test |
| | Trunk: 10.25 +/- 1.11 | 4 hearts | | 41 | | |
| 5A | Data analyzed from Asp et al. 2019 data set (neural crest cluster) | https://spatialtranscriptomics3d.shinyapps.io/Developmental_heart_e | N/A | N/A | N/A | N/A |
| 5B | Data from Litvinukova et al. 2020 data set (neuronal cell cluster) | https://www.heartcellatlas.org/ | N/A | N/A | N/A | N/A |
| 5C | Data analyzed from Skelly et al. 2018 data set | N/A | N/A | N/A | N/A | N/A |
| 5D | Data analyzed from Skelly et al. 2018 data set | N/A | N/A | N/A | N/A | N/A |
| | GFAP= 0.00% (main cluster), 1.8% (subcluster) | | | | | |
| | S100B= 3.40% (main cluster), 39.40% (subcluster) | | | | | |
| | Cdh11= 3.60% (main cluster), 36.70% (subcluster) | | | | | |
| | Id1= 20.50% (main cluster), 56.20% (subcluster) | | | | | |
| 5E | N/A | id1: 6 hearts | N/A | N/A | N/A | N/A |
| | N/A | cdh11: 6 hearts | N/A | N/A | N/A | N/A |
| 5F | Id1= 3.33 +/- 0.42 puncta | id1: 6 hearts | 12 | 1 per heart | N/A | N/A |
| | Cdh11= 2.33 +/- 0.33 puncta | cdh11: 6 hearts | | 1 per heart | | |
| 5G | Data analyzed from Asp et al. 2019 data set (neural crest cluster) | https://spatialtranscriptomics3d.shinyapps.io/Developmental_heart_e | N/A | N/A | N/A | N/A |
| | Data from Litvinukova et al. 2020 data set (neuronal cell cluster) | https://www.heartcellatlas.org/ | | | N/A | N/A |
| 6A | Data analyzed from Asp et al. 2019 data set (neural crest cluster) | https://spatialtranscriptomics3d.shinyapps.io/Developmental_heart_e | N/A | N/A | N/A | N/A |
| 6B | Data from Litvinukova et al. 2020 data set (neuronal cell cluster) | https://www.heartcellatlas.org/ | N/A | N/A | N/A | N/A |
| 6C | Data from Litvinukova et al. 2020 data set (neuronal cell cluster) | https://www.heartcellatlas.org/ | N/A | N/A | N/A | N/A |
| 6D | N/A | Brain: 5 hearts, Retina: 5 hearts | N/A | N/A | N/A | N/A |
| 6E | N/A | 6 hearts | N/A | N/A | N/A | N/A |
| 6F | Meteorin= 4.33 +/- 0.42 puncta | 6 hearts | 6 | 1 per heart | N/A | N/A |
| 6G | 2 dpf= 9.50 puncta | 2 dpf: 4 hearts | N/A | N/A | 2 vs 4: p=0.0027 | One-way ANOVA followed by Tukey's post hoc test |
| | 4 dpf= 47.00 puncta | 4 dpf: 5 hearts | | | 4 vs 6: p<0.0001 | One-way ANOVA followed by Tukey's post hoc test |
| | 6 dpf= 133.00 puncta | 6 dpf: 5 hearts | | | 2 vs 4 vs 6: p<0.0001 | One-Way ANOVA |
| 6H | 2 dpf: OT= 71.50%, Ventricle=26.84%, Atrium= 1.67% | 2 dpf: 3 hearts | N/A | N/A | N/A | N/A |
| | 4 dpf= OT= 33.49%, Ventricle=58.33%, Atrium= 8.18% | 4 dpf: 3 hearts | | | | |
| | 6 dpf= OT= 28.01%, Ventricle=62.24%, Atrium= 9.75% | 6 dpf: 3 hearts | | | | |
| 6I | N/A | N/A | N/A | N/A | N/A | N/A |
| 7A | N/A | N/A | N/A | N/A | N/A | N/A |
| 7B | N/A | metrn: 5 hearts, id1: 6 hearts, cdh11: 4 hearts | N/A | N/A | N/A | N/A |
| 7C | 6 mpf: 100% (wildtype), 100% (metrn+/-) | 6 mpf: (wildype: 10 alive, 0 dead) (metrn+/-: 6 alive, 0 dead) | N/A | N/A | N/A | N/A |
| | 9 mpf: 90.00% (wildtype) 66.67% (metrn +/-) | 9 mpf: (wildtype: 9 alive, 1 dead)(metrn+/-: 4 alive, 2 dead) | | | | |
| | 12 mpf: 80.00% 33.33% (metrn +/-) | 12 mpf: (wildtype: 8 alive, 2 dead)(metrn+/-: 2 alive, 4 dead) | | | | |
| 7D | N/A | Wildtype: 14 hearts, metrn-/-: 16 hearts | N/A | N/A | N/A | N/A |
| 7E | Wildtype: 10.29 +/- 1.05 cells | 14 hearts | 567 | 144 | All groups: p<0.0001 | One-Way ANOVA |
| | metrn+/-: 3.75+/- 0.80 cells | 20 hearts | | 75 | Wildtype vs metrn +/-: p<0.0001 | One-way ANOVA followed by Tukey's post hoc test |
| | metrn -/-: 3.75 +/- 0.86 cells | 16 hearts | | 60 | Wildtype vs metrn -/-: p<0.0001 | One-way ANOVA followed by Tukey's post hoc test |
| | metrn -/- + S31-201: 2.00 +/- 0.58 cells | 3 hearts | | 6 | metrn -/- vs metrn -/- + s31-201: p=0.9968 | One-way ANOVA followed by Tukey's post hoc test |
| | DMSO: 11.83 +/- 1.45 cells | 6 hearts | | 71 | | |
| | S31-201: 1.00 +/- 0.37 cells | 6 hearts | | 6 | DMSO vs S31-201: p<0.0001 | One-way ANOVA followed by Tukey's post hoc test |
| | DMSO: 11.00 +/- 1.37 cells | 6 hearts | | 66 | | |
| | CAS 457081-03-7: 0.83 +/- 0.31 cells | 6 hearts | | 5 | DMSO vs CAS: p<0.0001 | One-way ANOVA followed by Tukey's post hoc test |
| | DMSO: 9.17 +/- 0.79 cells | 6 hearts | | 55 | | |
| | Sc144: 13.17 +/- 1.35 cells | 6 hearts | | 79 | DMSO vs Sc144: p=0.5014 | One-way ANOVA followed by Tukey's post hoc test |
| 7F | 3 dpf: Wash-In = 0.40 +/- 0.24 nuclei, Wash-Out = 10.20 +/- 0.86 nuclei | 5 hearts per group | 165 | Wash in = 2, Wash out = | N/A | N/A |
| | 4 dpf: Wash-In = 2.40 +/- 0.51 nuclei, Wash-Out = 8.00 +/- 0.84 nuclei | 5 hearts per group | | Wash in = 12, Wash out | N/A | N/A |
| | 3 dpf: Wash-In = 9.80 +/- 0.86 nuclei, Wash-Out = 2.2+/- 0.66 nuclei | 5 hearts per group | | Wash in = 49, Wash out | N/A | N/A |
| 8A | DMSO: 129.40 +/- 1.33 bpm, MTZ: 155.10 +/- 1.50 bpm | DMSO: 7 hearts, MTZ: 7 hearts | N/A | N/A | DMSO vs MTZ: p<0.0001 | One-way ANOVA followed by Tukey's post hoc test |
| | Control Ablation: 131.4 +/- 1.15 bpm, Laser Ablation: 153.3 +/- 2.20 bpm | Control Ablation: 7 hearts, Laser Ablation: 7 hearts | N/A | N/A | Control Ablation vs Laser Ablation: p<0.0001 | One-way ANOVA followed by Tukey's post hoc test |
| | WT: 130.90 +/- 0.94 bpm, metrn +/-: 142.00 +/- 2.03 bpm, metrn -/-: 133.58 +/- 2.25 bpm | WT: 14 hearts , metrn +/-: 21 hearts, metrn -/-: 14 hearts | N/A | N/A | WT vs metrn +/-: p=0.0002, Wt vs metrn: p=0.9756 | One-way ANOVA followed by Tukey's post hoc test |
| | DMSO: 130.90 +/- 0.80 bpm, S31-201: 146.40 +/- 2.90 bpm | DMSO: 7 hearts, S31-201: 7 hearts | N/A | N/A | DMSO vs S31-201: p=0.0011 | One-way ANOVA followed by Tukey's post hoc test |
| | | | | | All groups: p<0.0001 | One-Way ANOVA |
| 8B | Wildtype: 1.08 +/- 0.53% fibrillation | 5 hearts | N/A | N/A | All groups: p=0.0008 | One-Way ANOVA |
| | metrn +/-: 16.39 +/- 4.81% fibrillation | 10 hearts | | | wildtype vs metrn +/-: p=0.1391 | One-way ANOVA followed by Tukey's post hoc test |
| | metrn -/-: 29.07 +/- 6.39% fibrillation | 6 hearts | | | wildtype vs metrn -/-: p=0.0037 | One-way ANOVA followed by Tukey's post hoc test |
| | Control Ablation: 0.26 +/- 0.26% fibrillation | 5 hearts | | | Control Ablation vs Laser Ablation: p=0.9708 | One-way ANOVA followed by Tukey's post hoc test |
| | Laser Ablation: 4.77 +/- 2.52% fibrillation | 5 hearts | | | | |
| 8C | N/A | 4 hearts | N/A | N/A | N/A | N/A |
| 8D | N/A | 4 hearts | N/A | N/A | N/A | N/A |
| 8E | N/A | 4 hearts | N/A | N/A | N/A | N/A |
| 8F | N/A | N/A | N/A | N/A | N/A | N/A |
| 8G | Control: 129.20 +/- 1.88 bpm | 5 hearts | N/A | N/A | All groups: p<0.0001 | One-Way ANOVA |
| | Full Ablation: 151.80 +/- 1.50 bpm | 5 hearts | N/A | N/A | Control vs Full: p<0.0001 | One-way ANOVA followed by Tukey's post hoc test |
| | Outflow Tract Ablation: 152.00 +/- 2.04 bpm | 4 hearts | N/A | N/A | Full vs OT: p>0.9999 | One-way ANOVA followed by Tukey's post hoc test |
| | Ventricular Ablation: 143.30 +/- 3.30 bpm | 4 hearts | N/A | N/A | Full vs V: p=0.1340 | One-way ANOVA followed by Tukey's post hoc test |
| | Atrium Ablation: 134.50 +/- 3.428 bpm | 4 hearts | N/A | N/A | Full vs A: p=0.0008 | One-way ANOVA followed by Tukey's post hoc test |
| 9A | N/A | 4 hearts | N/A | N/A | N/A | N/A |
| 9B | DMSO: 126.10 +/- 0.96 bpm | 7 hearts | N/A | N/A | All groups: p<0.0001 | One-Way ANOVA |
| | Baseline Isoproterenol: 157.60 +/- 1.00 bpm | 7 hearts | N/A | N/A | DMSO vs Iso: p<0.0001 | One-way ANOVA followed by Tukey's post hoc test |
| | Plus Full Ablation: 132.60 +/- 1.69 bpm | 5 hearts | N/A | N/A | Iso vs Full: p<0.0001 | One-way ANOVA followed by Tukey's post hoc test |
| | Plus Outflow Tract Ablation: 129.60 +/- 2.11 bpm | 5 hearts | N/A | N/A | Iso vs OT: p<0.0001 | One-way ANOVA followed by Tukey's post hoc test |
| | Plus Ventricular Ablation: 153.70 +/- 2.51 bpm | 6 hearts | N/A | N/A | Iso vs V: p=0.8249 | One-way ANOVA followed by Tukey's post hoc test |
| | Plus Atrium Ablation: 157.20 +/- 2.22 bpm | 5 hearts | N/A | N/A | Iso vs A: p>0.9999 | One-way ANOVA followed by Tukey's post hoc test |
| | Plus metrn -/-: 113.60 +/- 4.37 bpm | 5 hearts | N/A | N/A | Iso vs metrn -/-: p<0.0001 | One-way ANOVA followed by Tukey's post hoc test |
| 9C | DMSO: 130.60 +/- 1.81 bpm | 5 hearts | N/A | N/A | All groups: p<0.0001 | One-Way ANOVA |
| | Baseline Carbachol: 54.29 +/- 5.02 bpm | 7 hearts | N/A | N/A | DMSO vs Carb: p<0.0001 | One-way ANOVA followed by Tukey's post hoc test |
| | Plus Full Ablation: 147.20 +/- 4.10 bpm | 5 hearts | N/A | N/A | Carb vs Full: p<0.0001 | One-way ANOVA followed by Tukey's post hoc test |
| | Plus Outflow Tract Ablation: 117.60 +/- 3.02 bpm | 7 hearts | N/A | N/A | Carb vs OT: p<0.0001 | One-way ANOVA followed by Tukey's post hoc test |
| | Plus Ventricular Ablation: 95.40 +/- 5.14 bpm | 5 hearts | N/A | N/A | Carb vs V: p=0.0006 | One-way ANOVA followed by Tukey's post hoc test |
| | Plus Atrium Ablation: 68.50 +/- 5.24 bpm | 4 hearts | N/A | N/A | Carb vs A: p=0.6966 | One-way ANOVA followed by Tukey's post hoc test |
| | Plus metrn -/-: 104.80 +/- 17.07 bpm | 4 hearts | N/A | N/A | Carb vs metrn -/-: p<0.0001 | One-way ANOVA followed by Tukey's post hoc test |
| 9D | metrn -/- + DMSO: 26.50 +/- 5.25% fibrillation | 5 hearts | N/A | N/A | All groups: p<0.0001 | One-Way ANOVA |
| | Baseline Isoproternol: 8.18 +/- 1.98% fibrillation | 5 hearts | N/A | N/A | Baselin Isoproternol vs metrn -/- + Isoproternol: p<0.0001 | One-way ANOVA followed by Tukey's post hoc test |
| | metrn -/- + Isoproternol: 62.55 +/- 7.50% fibrillation | 5 hearts | N/A | N/A | Baseline carbachol vs metrn -/- + carbachol: p=0.8944 | One-way ANOVA followed by Tukey's post hoc test |
| | Baseline Carbachol: 1.62 +/- 0.72% fibrillation | 5 hearts | N/A | N/A | metrn-/- DMSO vs metrn-/- + isoproternol: p=0.0003 | One-way ANOVA followed by Tukey's post hoc test |
| | metrn -/- + Carbachol: 9.84 +/- 4.56% fibrillation | 5 hearts | N/A | N/A | metrn-/- DMSO vs metrn-/- + carbachol: p=0.2373 | One-way ANOVA followed by Tukey's post hoc test |
| 9E | N/A | N/A | N/A | N/A | N/A | N/A |
| S1-C | (zebrafish) N/A | 6 hearts | N/A | N/A | N/A | N/A |
| S1-D | (mouse) N/A | 10 hearts | N/A | N/A | N/A | N/A |
| S2-A | N/A | N/A | N/A | N/A | N/A | N/A |
| S2-B | Circularity: Wildtype= 0.8072, metrn -/-= 0.8042 | 5 | N/A | N/A | Wildtype vs metrn -/-: p=0.8792 | Unpaired t-test |
| | Roundness: Wildtype= 0.6670, metrn -/-= 0.6486 | | | | Wildtype vs metrn -/-: p=0.6774 | |
| | Solidity: Wildtype= 0.9710, metrn -/-= 0.9784 | | | | Wildtype vs metrn -/-: p=0.1362 | |
| | Aspect Ratior: Wildtype= 1.473, metrn -/-= 1.536 | | | | Wildtype vs metrn -/-: p=0.5643 | |
| S2-C | Circularity: Wildtype= 0.8954, metrn -/-= 0.8874 | 5 | N/A | N/A | Wildtype vs metrn -/-: p=0.5900 | Unpaired t-test |
| | Roundness: Wildtype= 0.6798 , metrn -/-= 0.6542 | | | | Wildtype vs metrn -/-: p=0.5392 | |
| | Solidity: Wildtype= 0.9878, metrn -/-= 0.9852 | | | | Wildtype vs metrn -/-: p=0.3950 | |
| | Aspect Ratior: Wildtype= 1.491, metrn -/-= 1.540 | | | | Wildtype vs metrn -/-: p=0.6065 | |
| S2-D | Circularity: Wildtype= 0.8562, metrn -/-= 0.8308 | 5 | N/A | N/A | Wildtype vs metrn -/-: p=0.0779 | Unpaired t-test |
| | Roundness: Wildtype= 0.6516, metrn -/-= 0.6366 | | | | Wildtype vs metrn -/-: p=0.7138 | |
| | Solidity: Wildtype= 0.9764, metrn -/-= 0.9750 | | | | Wildtype vs metrn -/-: p=0.7968 | |
| | Aspect Ratior: Wildtype= 1.611, metrn -/-= 1.605 | | | | Wildtype vs metrn -/-: p=0.8837 | |
